# Endosperm culture-based allotriploid hybrid production from an interspecific cross of *Haemanthus* spp.: new insights into polyploidization and hybridization

**DOI:** 10.1186/s12870-025-06181-x

**Published:** 2025-02-06

**Authors:** Arisa Nakano, Masahiro Mii, Yoichiro Hoshino

**Affiliations:** 1 Field Science Center for Northern Biosphere, Hokkaido University, Kita 11, Nishi 10, Kita-Ku, Sapporo, 060-0811 Japan; 2 Center for Environment, Health and Field Sciences, Chiba University, Kashiwanoha 6-2-1, Kashiwa, Chiba, 277-0882 Japan

**Keywords:** Allopolyploid, Cytoplasmic inheritance, Embryo, Endosperm, *Haemanthus*, Hybridization, Plant tissue culture, Regeneration, Triploid

## Abstract

**Background:**

Allopolyploid plants are valuable for plant breeding because they have the advantage of polyploidization and hybridization, such as increased vigor and adaptability. Although biparental triploid endosperms have the potential to be used to produce allotriploid plants, the approach remains largely unexplored. Therefore, this study aimed to produce allotriploid plants from the endosperms of interspecific crosses between *Haemanthus pauculifolius* and *H. albiflos*.

**Results:**

Precisely identified embryo and endosperm pairs were used. Embryos were grown on half-strength Murashige and Skoog (MS) medium, and endosperms from interspecific crossing were cultured to induce callus formation and shoot regeneration, which then developed into plantlets. MS medium supplemented with 4-amino-3,5,6-trichloropicolinic acid (picloram) and 6-benzylaminopurine (BAP), or 2,4-dichloro phenoxy acetic acid (2,4-D) and BAP were used for callus induction, and callus formation rates were measured. Flow cytometry, karyotyping, and Sanger sequencing of the nuclear internal transcribed spacer (ITS) region, chloroplast (*trnL-trnF* region, *matK* gene), and mitochondrial (*nad1* gene) DNA were performed on plantlets derived from embryos and endosperms, along with their parental plants. In this study, a total of 18 pairs of diploid and triploid plantlets were obtained from the embryo and endosperm, respectively. Callus formation rates were significantly higher on media with picloram and BAP compared to 2,4-D and BAP. ITS sequencing and karyotype analyses detected that all the 16 pairs of plantlets analyzed were hybrids, indicating that most endosperm-derived plantlets were allotriploid with a parental chromosome ratio of 2:1 (maternal: paternal). In addition, chloroplast DNA sequencing revealed maternal inheritance in the endosperm-derived plantlets, consistent with embryo-derived plantlets.

**Conclusions:**

This study is the first to demonstrate the production of allotriploid hybrid plants through endosperm culture using seeds from interspecific crosses, as supported by cellular and genetic analyses. Additionally, the study established a novel system for simultaneously producing diploid and allotriploid hybrids from a single seed, providing valuable materials to study the effects of polyploidization and hybridization in allopolyploid plants. These findings contribute to plant breeding strategies and advance our understanding of hybridization, polyploidization, and allopolyploid plant development.

**Supplementary Information:**

The online version contains supplementary material available at 10.1186/s12870-025-06181-x.

## Background

Polyploidy is defined as the possession of three or more complete sets of chromosomes and has been recognized as one of the driving forces in the evolutionary process of vascular plants [1]. A polyploid individual that arises within or between populations of a single species is referred to as autopolyploid, whereas individuals of hybrid origin are termed allopolyploid [2]. Comai [3] described three advantages of polyploids, especially in allopolyploid plants. First, heterosis causes polyploids to be more vigorous than their diploid progenitors; second, gene redundancy shields polyploids from the deleterious effects of mutations; and third, asexual reproduction enables polyploids to reproduce in the absence of sexual mates. The most widespread consequence of polyploidy in plants is the increase in cell size, caused by the larger number of gene copies, referred to as the “gigas” effect [4]. In addition, some odd-numbered polyploid plants, such as triploids (3*x*), pentaploids (5*x*), and septaploids (7*x*), have sterile seeds or produce seedless fruits as a result of unbalanced meiosis. Seedlessness is used to improve the edible quality of fruits such as banana, apple, citrus, grape, and papaya; hence, synthetic triploid plant production is of immense importance [5]. Thus, polyploidization has been known to be one of the plant breeding strategies.

Colchicine, a naturally occurring alkaloid, is most frequently employed as an antimitotic agent to produce synthetic polyploid plants. It induces polyploid cell formation by disrupting microtubule formation during mitosis and arresting the cell cycle at metaphase [6]. This prevents chromosome pairs from separating, resulting in the formation of polyploid cells. Anti-mitotic herbicides such as oryzalin, trifluralin, flufenacet, a chemical mixture of amiprophosmethyl + pronamide + dimethyl sulfoxide, and nitrous oxide gas can be used instead of colchicine as less toxic alternatives to antimitotic agents [7]. Odd-numbered polyploid plants, especially triploid plants, can be produced by natural selection, sexual hybridization with unreduced gametes, interploid crossing between diploid and tetraploid plants, fusion of diploid somatic protoplasts and haploid microspore cells, and endosperm culture [8].

Endosperm is a distinct tissue acquired by angiosperms during their evolution. Most angiosperms have a *Polygonum*-type embryo sac, which is composed of eight nuclei divided into seven cells: an uninucleate egg cell, a binucleate central cell, and five additional uninucleate cells of two synergids and three antipodals [9]. The central cell fuses with a one-nucleus sperm cell released from the pollen to form a triploid endosperm in diploid angiosperms. Plants can regenerate through somatic embryogenesis in vitro, in which isolated protoplasts or cells first develop into cellular structures that are similar to zygotic embryos before developing into whole plant bodies [10]. Attempts to culture endosperm tissue began in the early 1930s by Lampe and Mills, and since then, the method has been known to offer a one-step approach to triploid production as opposed to conventional methods, which involve hybridization between tetraploids and diploids [5].

Polyploidization is an effective and common approach to plant breeding. Production has improved and become efficient in autopolyploid plants, even in odd-numbered polyploid plants. However, barriers still exist to the production of allopolyploid plants, even though they have desirable traits. Endosperm is a biparental and polyploid tissue that has the potential to produce allopolyploid plants directly from diploid plants. However, most previous research on endosperm culture for plant regeneration focused on the effective production of autopolyploid plants; the endosperms used as explants were derived from open-pollination [11, 12], self-pollination [13–16], and intraspecific cross to avoid self-incompatibility [17]. Although a few endosperm cultures along with an interspecific cross in *Actinidia* species [18] and *Citrus* species [11] and an intergeneric cross between wheat and rye [19] have been reported; these endosperm-derived plants have been suggested to be hybrids based on crossing method and leaf morphology. In addition, the inheritance of chloroplast DNA (cpDNA) and mitochondrial DNA (mtDNA) from cytoplasmic DNAs of endosperm-derived plants remains unclear. Therefore, it is necessary to determine whether endosperm culture is available to produce hybrids (allopolyploids) with their parents by comparing the DNA content and performing karyotype and genetic analyses.

The genus *Haemanthus*, belonging to the Family Amaryllidaceae, is a mainly deciduous, autumn-flowering and winter-growing group of bulbous geophytes confined to southern African countries [20]. The genus is composed of 23 species, that is, 21 species reported by Snijman [21], followed by *Haemanthus pauculifolius* in 1993 [22], and *Haemanthus humanii* in 2022 [20]. The basic chromosome number and ploidy levels have been reported to be *n* = 8 and diploid [23]. *Haemanthus albiflos* Jacq. and *H*. *pauculifolius* most closely resemble the four evergreen species with white-colored flowers, but the pattern of leaf growth and floral dimensions are different. *Haemanthus pauculifolius* has velutinous leaves on both surfaces and fewer leaves and flowers than *H*. *albiflos* [22]. We previously investigated the production of various autopolyploid plants from diploid *H*. *albiflos* using colchicine treatment combined with embryo and endosperm culture and found that the endosperm of *H. albiflos* was stable and exhibited high callus induction and plant regeneration capacity compared to other plant species [16, 24].

The objective of the present study was to simultaneously produce diploid and triploid (allotriploid) hybrid plants from the embryo and endosperm pairs of seeds derived from an interspecific cross between *H. pauculifolius* and *H*. *albiflos*. Flow cytometry, internal transcribed spacer (ITS) sequencing, and karyotype analyses were conducted to confirm the origin and investigate whether the produced plantlets were hybrids. Furthermore, the inheritance pathway was investigated by sequencing the *trnL*-*trnF* region, *matK* gene, and *nad1* gene of the cytoplasmic genomes in endosperm-derived plantlets and compared them with those of their parents and the embryo-derived plantlets. These findings will contribute to efficient plant breeding and investigation of the effects of allopolyploidization, including hybridization and polyploidization.

## Methods

### Plant materials and interspecific cross

**Fig. 1:**
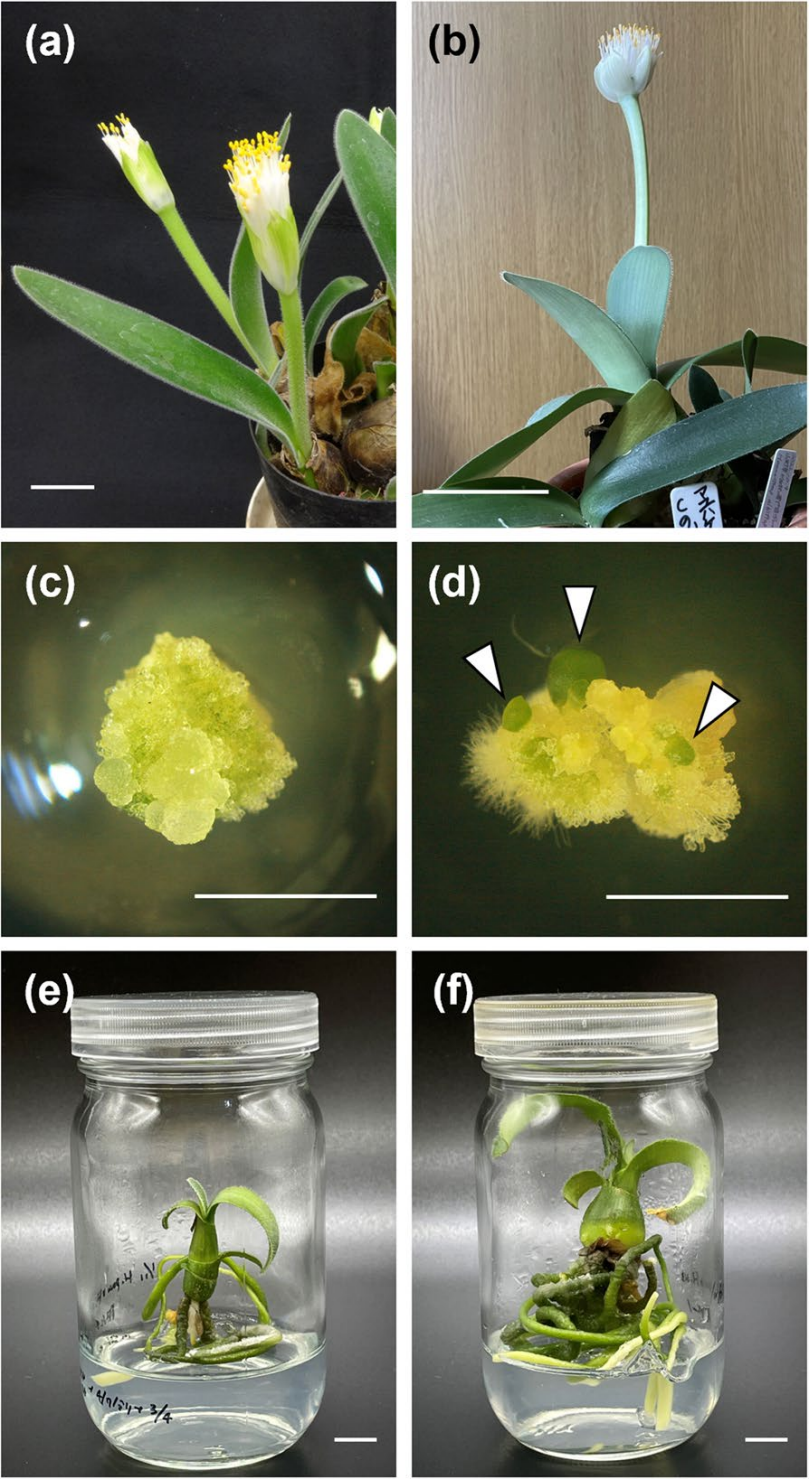
Images of *Haemanthus pauculifolius*, *H*. *albiflos*, endosperm culture-derived cultures and plantlets, and embryo culture-derived plantlets. **a** *H*. *pauculifolius* (seed parent). **b** *H*. *albiflos* (pollen parent). **c** Callus at four weeks after culture (WAC) of endosperm. **d** Callus-produced shoots on the regeneration medium at four WAC. Arrowheads indicate shoots. **e** Endosperm culture-derived plantlet (EN4-1). **f** Embryo culture-derived plantlet (EM4-1). Scale bars: 5 cm (**a**, **b**), 0.5 cm (**c**, **d**), and 1 cm (**e**, **f**)

Diploid plants of *Haemanthus pauculifolius* Snijman & van Wyk (2*n* = 2*x* = 16) (Fig. 1a) and *Haemanthus albiflos* Jacq. (2*n* = 2*x* = 16) (Fig. 1b) were used in this study. These plants were obtained from a commercial nursery and subsequently cultivated in a greenhouse at Hokkaido University, Hokkaido, Japan, under natural light and automatic ventilation, with the temperature maintained at approximately 25 °C. Interspecific crossing was conducted using *H*. *pauculifolius* as the seed parent and *H*. *albiflos* as the pollen parent. The anthers of *H*. *albiflos* were collected using tweezers and stored at 4 °C until use. After emasculation, the pollen grains from the anthers were placed on the stigmas of *H*. *pauculifolius* to complete the interspecific crossing until all the flowers in the umbel were opened. Twelve green-colored immature ovaries containing 22 seeds were collected at nine weeks and two days after the first pollination. The seeds removed from the ovaries were surface-sterilized with sodium hypochlorite (1% active chlorine); rinsed with sterilized distilled water; cut into longitudinal sections; and separated into embryo, endosperm, and seed coat using a scalpel and tweezers in a glass dish on a clean bench. The embryo and endosperm were used for each culture process.

### Embryo culture

Twenty-two embryos were cultured in half-strength Murashige and Skoog (MS) medium [25]. The MS medium contained 30 g L^−1^ sucrose and 3 g L^−1^ gellan gum, and the pH was adjusted to 5.7 ± 0.1 before autoclaving at 121 °C for 15 min. The cultures were incubated at 25 °C under 24-h continuous light.

### Endosperm culture

Twenty-two endosperms were used for endosperm culture according to the procedure by Nakano et al*.* [16], with minor modifications. Briefly, the endosperm was cut into eight pieces, and four pieces, including the chalazal and micropylar poles, were cultured on MS medium with different combinations of plant growth regulators (PGRs): 1) 5 mg L^−1^ of 4-amino-3,5,6-trichloropicolinic acid (picloram) and 5 mg L^−1^ of 6-benzylaminopurine (BAP) and 2) 5 mg L^−1^ of 2,4-dichloro phenoxy acetic acid (2,4-D) and 5 mg L^−1^ of BAP. A total of 86 and 87 pieces were cultured in the respective media. The cultures were incubated at 25 °C under 24-h continuous light, and the callus formation frequency was recorded at four and eight weeks after culture (WAC). Three media were prepared to induce shoot regeneration from the calli: 1) half-strength MS medium without plant growth regulators, 2) MS medium without plant growth regulators, 3) MS medium supplemented with 0.5 mg L^−1^ potassium naphthalene-1-acetate (NAA) and 1 mg L^−1^ BAP. These media contained 30 g L^−1^ sucrose and 3 g L^−1^ gellan gum. After eight weeks of culture on the medium for callus induction, calli were cut into three pieces with a scalpel and tweezers and transferred onto each medium. Shoots that regenerated from the calli were transferred onto the half-strength MS medium without plant growth regulators and grown into plantlets.

### Flow cytometric analysis

Flow cytometric analysis of plantlets produced from embryo and endosperm cultures was conducted as described in Nakano and Hoshino [24] to detect each ploidy level and the original tissue. Briefly, plantlet leaves and an internal standard, *Zephyranthes candida* (Lindl.) Herb, were cut into approximately 0.5 cm square. The leaf sample was chopped with a razor blade in 200 µL of ice-cold nuclei extraction buffer (Quantum Stain NA UV2, Quantum Analysis, Münster, Germany). The sample solution was filtered through with a 30 µm nylon mesh into a sample tube, and 800 µL of ice-cold solution containing 10 mM of Tris, 50 mM of sodium citrate, 2 mM of MgCl_2_, 1% (w/v) of Triton X-100, and 2 mg L^−1^ of 4′6-diamino-2-phenylindole (DAPI) (pH 7.5) [26] was added. The relative fluorescence intensities of the samples were measured using a flow cytometer (Ploidy Analyzer PA; Partec, Münster, Germany). In this study, the fluorescence intensity of the internal standard was set to 50.

### Internal transcribed spacer (ITS) sequencing

The ITS regions of the plantlets were sequenced and compared to determine whether the plantlets were hybrids. Crude DNA of *H*. *pauculifolius*, *H*. *albiflos*, the 21 embryo-derived plantlets, and the 16 endosperm-derived plantlets were extracted from the leaf segment according to the one-step method described in the instruction of KOD One® (TOYOBO CO., LTD, Osaka, Japan). Primers for amplification and sequencing and ITS4 and ITS5 [27] were selected from the primer list (Table S1) described in Rønsted et al*.* [28]. The polymerase chain reaction (PCR) reaction mix (50 µL) contained a polymerase (1U KAPA2G Robust HotStart ReadyMix, Kapa Biosystems, Roche, Basel, Switzerland) with 5 µL of 5 μM of each primer and 1 μL DNA template. PCR was performed using a thermal cycler (GeneExplorer Thermal Cycler GE-96G, Hangzhou Bioer Technology Co. Ltd., Hangzhou, China) with the following parameters: 3 min at 95 °C, followed by 40 cycles and 15 s at 95 °C, 30 s at 56 °C, and 60 s at 72 °C, followed by a final extension step of 60 s at 72 °C. The PCR products were electrophoresed on 1% agarose gels and stained with ethidium bromide to confirm the amplification and size of the DNA fragments. The DNA fragments were purified using the ethanol precipitation method and dissolved in sterile water. After purification, the quality and quantity were measured using NanoDrop® ND-1000 Spectrophotometer (Thermo Fisher Scientific, MA, USA), and Sanger sequencings were performed by GENEWIZ (Azenta Life Sciences, Tokyo, Japan). The sequences were aligned to the sequence data (Table S2) of the ITS region of *Haemanthus* species registered in the National Center for Biotechnology Information (NCBI) database to confirm whether the desired area was acquired and analyzed using the online Benchling software (https://www.benchling.com/).

### Chromosome observation and karyotype analysis

The numbers of *H*. *pauculifolius*, *H*. *albiflos*, the 21 embryo-derived plantlets, and the 16 endosperm-derived plantlets chromosomes were counted to detect whether the plantlets were hybrids. Pretreatment and fixation were performed as described in Tanaka and Taniguchi [29] with minor modifications. The root tips of approximately 0.5–1.0 cm in length were cut out from the plantlet and soaked in 2 mM of 8-hydroxyquinoline for 4 h at ambient temperature in dark conditions. The root tips were fixed in ethanol-acetic acid (3:1) for 1 h at 4 °C. Enzymatic maceration/air-drying method was performed as described in Fukui and Iijima [30] with minor modifications. Briefly, the fixed root tips were washed in distilled water for 30 min at ambient temperature. The root tips were macerated with a 50 µl of enzyme mixture of 2% Cellulase Onozuka R-10 (Yakult Pharmaceutical Co., Ltd., Tokyo, Japan) and 1% Pectolyase Y23 (Seishin Pharmaceutical Co., Ltd., Tokyo, Japan) in microtubes at an incubator set to 37 °C for 65 min. The meristematic portions of the root tips were removed by pipetting with a Pasteur pipette, placed into distilled water in a Petri dish, and washed for 3 min. The meristematic portions were transferred onto a glass slide, covered with few drops of ethanol:acetic acid (3:1), and tapped with the tip of the forceps until they were invisible. After air-drying overnight, drops of acetic orcein solution were applied to the glass slide and covered with a coverslip. Chromosome images were captured using an upright microscope (AxioImager M1, Carl Zeiss, Oberkochen, Germany) coupled to a digital camera (AxioCam MRm; Carl Zeiss) and the ZEISS ZEN 3.7 (blue edition) software (Carl Zeiss). Clear images depicting individual chromosomes were used for chromosome identification. Chromosome observation and karyotyping of each parent, embryo- and endosperm-derived plantlet were conducted in at least three cells to detect their chromosome numbers and karyotypes.

### Sequencing of the cytoplasmic genomes

To investigate the inheritance of cytoplasmic genomes in endosperm- and embryo-derived plantlets, the maturase K (*matK*) gene and *trnL-trnF* region of cpDNA and NADH dehydrogenase subunit 1 (*nad1*) gene of mtDNA were sequenced and compared. Crude DNA samples from *H*. *pauculifolius*, *H*. *albiflos*, and six pairs of embryo- and endosperm-derived plantlets were used. The pairs were selected based on the ITS sequencing results. Primers for amplification and sequencing, 19F [31] and 2R [32] for *matK* gene, trnC and trnF [33] for *trnL-trnF* region, and nad1eB and nad1eCR [34] for *nad1* gene were selected from the primer list (Table S1) described in Rønsted et al*.* [28]. The 50 µL PCR reaction mix for *matK* and *trnL-trnF* contained the polymerase with 5 µL of 5 μM of each primer and 1 μL of DNA template. PCR was performed with the following parameters: 3 min at 95 °C, followed by 38 cycles and 15 s at 95 °C, 30 s at 47 °C, and 100 s at 72 °C, followed by a final extension step of 100 s at 72 °C. The PCR reaction mix for nad1 was changed in the DNA template to 2 µL and the annealing temperature to 51 °C. After confirming the amplification and size of the DNA fragments in the PCR products, the DNA fragments were purified using the ethanol precipitation method and dissolved, and their quality and quantity were measured. In addition to the above primers, the Sanger sequencings were performed using primers KatF [35] and 1326R [36] for *matK* gene and nad1iB2 and nad1iB2R [37] for *nad1* gene selected from the primers described in Rønsted et al*.* [28]. The sequences were aligned to the sequence data (Table S2) of the *trnL-trnF* region, *matK* gene, and *nad1* gene of *Haemanthus* species registered in NCBI to confirm whether the desired area was acquired and analyzed.

### Statistical analysis

The differences in callus formation rates from endosperm pieces between the two callus induction media at each measurement time (four WAC, eight WAC, and last) were analyzed using a chi-square test with a significance level of *p* < 0.05.

## Results

### Endosperm and embryo culture

In endosperm culture, callus formation was observed on both media for callus induction (Table 1, Table S3, Fig. 1c). Eight seeds (36.4%) and five seeds (22.7%) formed calli at four WAC, with no significant difference observed. Callus formation at eight WAC was observed on the medium supplemented with picloram and BAP in 18 seeds (81.8%), which was significantly higher than 11 seeds (50.0%) of the medium with 2,4-D and BAP. This trend continued to the final measurement. Endosperm pieces of four seeds (EN2-3, EN10-1, EN10-2, and EN10-3) did not form calli on either medium. After the transfer of calli onto the medium for shoot regeneration, the calli, which were induced on the medium supplemented with picloram and BAP, regenerated shoots on at least one or more media (Tables 1 and S3, Fig. 1d). Shoot regeneration was observed in seven endosperms on the calli formed from the 12 endosperms cultured on the medium supplemented with 2,4-D and BAP until eight WAC. The shoots were transferred to the half-strength MS medium and grown into plantlets (Fig. 1e). Among the 22 cultured embryos, 21 embryos, except for EM10-3, grew into plantlets (Table 1, Fig. 1f). Eighteen pairs of plantlets were obtained from the endosperm and embryo of the same seed. Although the plantlets of EN1-2 and EN11-2 were obtained, subsequent analyses could not be conducted because their date was later than that of others.

**Table 1:** Numbers and frequencies of the callus formation and plantlet regeneration in the cultures

Explant type	No. of explants (pieces)	Callus induction medium	Callus formation at 8WAC (%)	Regeneration medium	Plantlet regeneration per explants (%)
Embryo	22^a^	–^c^	–^c^	1/2MS HF	21 (95.5)
Endosperm	22^b^	MS	18 (81.8)	1/2MS HF	12 (66.7)
	(86)	Picloram + BAP	56* (65.1)	MS HF	13 (72.2)
				MS NAA + BAP	17 (94.4)
	22^b^	MS	14 (63.6)	1/2MS HF	6 (42.9)
	(87)	2,4-D + BAP	31* (35.6)	MS HF	6 (42.9)
				MS NAA + BAP	6 (42.9)

HF means PGR-free media. The detailed information was described in Table S3

^a^Whole embryos were used for the culture

^b^Endosperm pieces were used after cutting for the culture

^c^Not examined

*Significant differences in callus formation between the callus induction media were detected using a chi-square test at *p* < 0.05

### Flow cytometric analysis

**Fig. 2:**
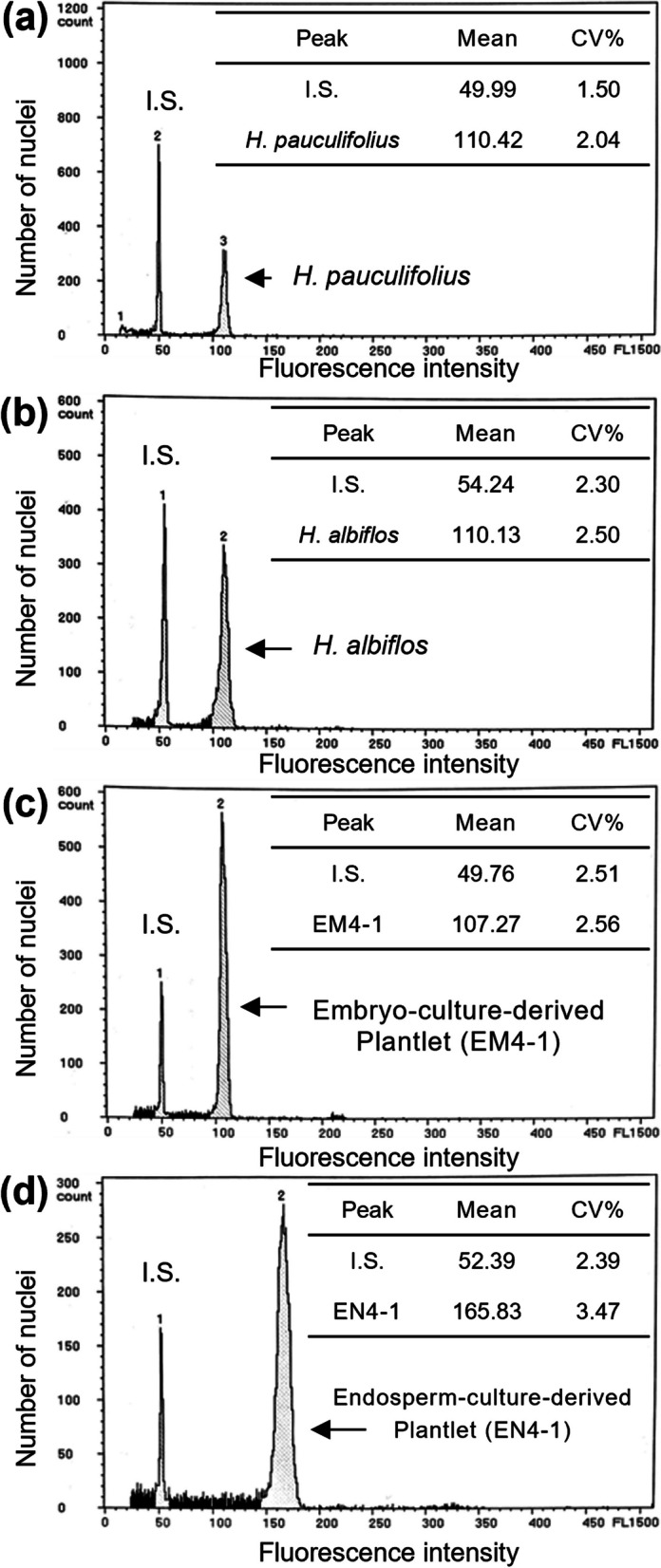
Representative histograms of relative DNA contents in the parents and the embryo- and endosperm-culture-derived plantlets. **a** *H*. *pauculifolius* used as the seed parent. **b** *H*. *albiflos* used as the pollen parent. **c** Embryo-culture-derived plantlet. **d** Endosperm-culture-derived plantlet. I.S. indicates that *Z*. *candida* was used as an internal standard

The average standard deviations of the fluorescence intensities of the diploid parents were 107.40 ± 3.90 in* H*. *pauculifolius* (Fig. 2a) and 105.25 ± 2.68 in *H*. *albiflos* (Fig. 2b) at the internal standard fluorescence intensity of 50; these intensities of the parents are close. Accordingly, this analysis was designed to detect the ploidy level, and was not intended to determine whether the plantlets are hybrids. In the 21 embryo-culture-derived plantlets, the fluorescence intensity was 108.08 ± 0.89 (Fig. 2c), which is equal to that of the diploid parent plants, indicating that they were all estimated to be diploid and originated from the embryo. In the 16 endosperm-culture-derived plantlets, except for EM1-2 and EM11-2, the average fluorescence intensity was 160.96 ± 3.34 (Fig. 2d), which is 1.5-fold that of the diploid parents, indicating that they were estimated to be triploid and originated from the triploid endosperm tissue.

### ITS sequencing

**Fig. 3:**
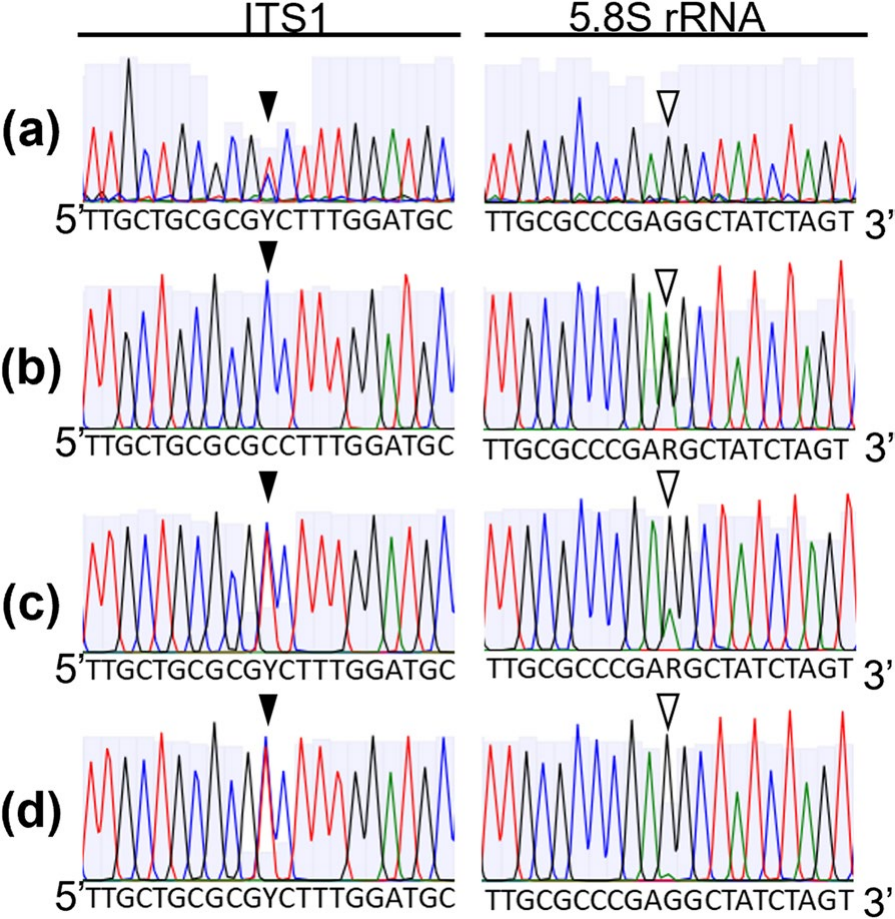
Sanger sequencing chromatograms of the ITS region of the parents and embryo- and endosperm-derived plantlets. **a** *H*. *pauculifolius* used as a seed parent. **b** *H*. *albiflos* used as a pollen parent. **c** Plantlets derived from the embryo. **d** Plantlets derived from the endosperm. Black arrowheads mean the nucleotide position in ITS1 region: *H*. *pauculifolius* carried a mixed nucleotide “Y” (T + C), and *H*. *albiflos* carried a “C.” White arrowheads mean the nucleotide position in 5.8S rRNA gene: *H*. *pauculifolius* carried a “G,” and *H*. *albiflos* carried a “R” (A + G)

Electrophoresis images showing the amplification of the ITS region are presented in Fig. S1. The sequencings of the ITS region, including the partial ITS1 region, 5.8S ribosomal RNA (rRNA) genome, and complete ITS2 region, were obtained, and two single nucleotide polymorphisms (SNPs) were identified. The SNP in ITS1 region was “Y” (C + T) and “C,” and the other in 5.8S rRNA was “G” and “R” (A + G) between *H*. *pauculifolius* (Fig. 3a) and *H*. *albiflos* (Fig. 3b), respectively. Two types of sequences were observed (Fig. 3c, d) in the plantlets. In 21 embryo-derived plantlets, all nucleotides were “Y” at SNP in the ITS1 region; eight plantlets were “R,” and 13 plantlets were “G” at SNP in the 5.8S rRNA (Table S4). In 16 endosperm-derived plantlets, all nucleotides were “Y” at SNP in the ITS1 region; four plantlets were “R,” and 12 plantlets were “G” at SNP in the 5.8S rRNA (Table S4). With two peaks, “A” and “G,” it was impossible to determine whether the “G” at SNP in the 5.8S rRNA was derived from the seed parent *H*. *pauculifolius* only or from both *H*. *pauculifolius* and the pollen parent *H*. *albiflos*; therefore, they could not be identified. In addition, if the embryo was a hybrid, its endosperm counterpart may be considered to be a hybrid. Thus, these results indicate that eight pairs of seeds in which the embryo-derived plantlets showed “R” in the 5.8S rRNA SNPs were at least seeds fertilized *H*. *pauculifolius* and *H*. *albiflos*.

### Chromosome observation and karyotype analysis

**Fig. 4:**
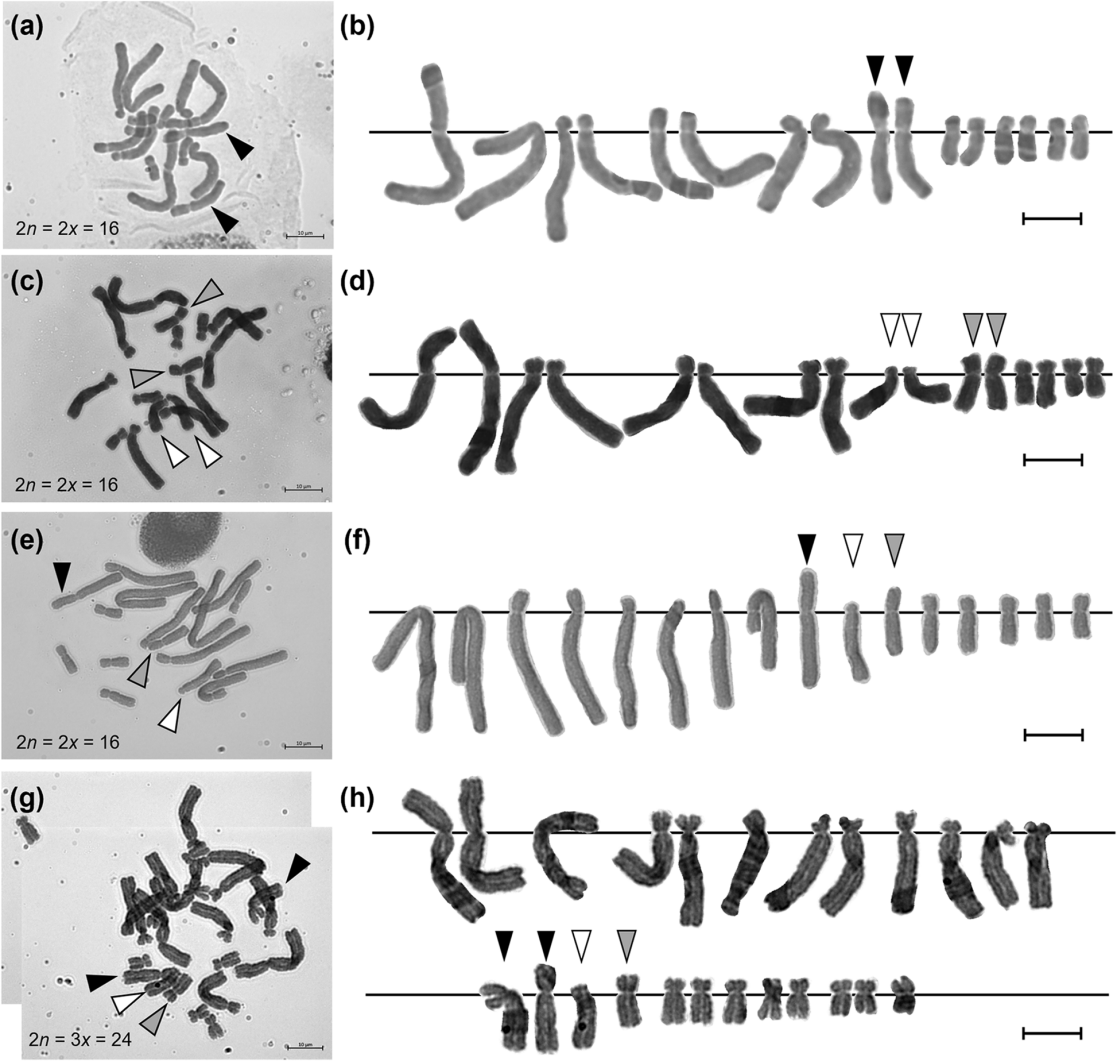
Representative chromosome images and karyograms of *Haemanthus pauculifolius*, *H*. *albiflos*, and embryo- and endosperm-derived plantlets. Images of chromosome (**a**) and karyogram (**b**) of diploid (2*n* = 2*x* = 16) *H*. *pauculifolius* used as a seed parent. Image of chromosome (**c**) and karyogram (**d**) of diploid (2*n* = 2*x* = 16) *H*. *albiflos* used as a pollen parent. Images of chromosome (**e**) and karyogram (**f**) of diploid (2*n* = 2*x* = 16) embryo-derived plantlet (EM2-1). Images of chromosome (**g**) and karyogram (**h**) of triploid (2*n* = 3*x* = 24) endosperm-derived plantlet (EN12-1). Each arrowhead means the characteristic chromosome of *H*. *pauculifolius* (black) and *H*. *albiflos* (white and gray). Scale bars = 10 µm

Chromosome observations revealed three distinctive chromosomes: one of *H*. *pauculifolius* and two of *H*. *albiflos*, which were unique in length and kinetochore position in the karyotype between *H*. *pauculifolius* (Fig. 4a, b) and *H*. *albiflos* (Fig. 4c, d). Karyotype analyses were conducted on these three chromosomes in the plantlets derived from the embryo and endosperm to detect whether they were hybrids. Chromosome observations revealed that the chromosome numbers of all the 21 embryo-derived plantlets were 16 (2*n* = 2*x* = 16), including each of the three chromosomes that we focused on (Fig. 4e, f, Fig. S2). The chromosome number of all the 16 endosperm-derived plantlets was 24 (2*n* = 3*x* = 24), except for EN3-1, which exhibited a reduced chromosome number. These endosperm-derived plantlets contained four chromosomes from the three chromosomes that we focused on: two homologous chromosomes of *H*. *pauculifolius* and two different chromosomes of *H*. *albiflos* (Fig. 4g, h, Fig. S3). These results indicate that the plantlets produced from the embryo and endosperm were hybrids of *H*. *pauculifolius* and *H*. *albiflos*.

### Sequencing of the cytoplasmic genomes

Electrophoresis images showing the amplification of the *trnL-trnF* region, *matK* gene, and *nad1* gene are presented in Fig. S4. In the sequences of *H*. *pauculifolius* and *H*. *albiflos*, two and three SNPs were found in the *trnL-trnF* region and *matK* gene of cpDNA, respectively, and no SNP were found in the *nad1* gene of mtDNA (Table 2, Fig. S4). The six pairs of embryo- and endosperm-derived plantlets had the same SNPs pattern as the seed parent, *H*. *pauculifolius*, suggesting that the inheritance of the chloroplast genome was maternal in both the embryo and the endosperm.

**Table 2:** The SNPs of cpDNA and mtDNA in parents and the embryo- and endosperm-derived plantlets

	cpDNA					mtDNA
	*trnL-trnF* region		*matK* gene			*nad1* gene
Sample ID	SNP#1	SNP#2	SNP#1	SNP#2	SNP#3	SNP
*H. pauculifolius*	A	T	C	T	A	–^a^
*H. albiflos*	C	C	T	C	G	–
EM1-1	A	T	C	T	A	–
EM4-1	A	T	C	T	A	–
EM6-1	A	T	C	T	A	–
EM7-2	A	T	C	T	A	–
EM8-1	A	T	C	T	A	–
EM11-1	A	T	C	T	A	–
EN1-1	A	T	C	T	A	–
EN4-1	A	T	C	T	A	–
EN6-1	A	T	C	T	A	–
EN7-2	A	T	C	T	A	–
EN8-1	A	T	C	T	A	–
EN11-1	A	T	C	T	A	–
Inheritance	Maternal	Maternal	Maternal	Maternal	Maternal	Not detected

*EM* embryo-derived plantlets, *EN* endosperm-derived plantlets

^a^No SNP was found in this study

## Discussion

In the present study, the endosperm from an interspecific cross between *H*. *pauculifolius* and *H*. *albiflos* was used to induce callus formation and shoot regeneration. Callus formation and subsequent shoot regeneration were both observed at a stable induction rate on MS medium supplemented with picloram and BAP, and this is similar to the previously reported self-pollination of *H*. *albiflos* [16]. The other medium supplemented with 2,4-D and BAP, which seemed to be a combination of the most effective auxin and popular cytokinin for callus formation from endosperm [8], showed a relatively lower frequency of callus formation and later shoot regeneration. A similar trend was observed in the genus *Actinidia* [18], which reported that a medium supplemented with 2,4-D promoted callus induction from the endosperm, but the calli were slower and more difficult to differentiate, especially in the endosperm from an interspecific cross. In addition, Kin et al*.* [18] reported a lack of a genotype-related influence on endosperm callus induction in the genus *Actinidia*. It has been reported that species, cultivars, and genotypes influence the responsiveness to endosperm culture in *Passiflora* species [38], *Malus pumila* [39], and *Lonicera caerulea* [17], respectively. These variations in plant regenerative abilities have been reported in several plant tissue cultures and are influenced by several factors, such as the use of PGRs, composition of the basic medium, explant type, and plant species [40]. In addition to these factors, the developmental stage of the endosperm is an important factor in endosperm culture [5, 41]. In interploid and interspecific hybridization, aberrant endosperm development resulting from the disruption of parental genome balance and genomic imprinting has been reported in both dicotyledonous plants, such as *Arabidopsis* [42, 43], and monocotyledonous plants, such as rice [44, 45]. Maternal genome excess, the cross between female tetraploids and male diploids, shows precocious endosperm cellularization, but paternal genome excess, the cross between female diploids and male tetraploids, shows delayed cellularization [42]. Therefore, aberrant endosperm development is predicted to affect plant regeneration. Although not observed in the interspecific cross between *H*. *pauculifolius* and *H*. *albiflos* in the present study, there remains a need to investigate the effects on the availability of endosperm for interspecific hybrid production in the future.

We used several approaches to confirm that the plantlets obtained from the endosperm cultures were allotriploid. Flow cytometric analysis and chromosome observations have been commonly conducted in previous studies on endosperm cultures to detect the ploidy levels of the produced plants, confirm the original tissue, and assess the stability of the original ploidy level during the culture process. Aneuploid and other ploidy levels have been found in endosperm-derived plants that are formed from even open- or self-pollination. In diploid *Actinidia chinensis*, most of the endosperm culture-derived plants were aneuploid, and a few were triploids [46], and mixoploid chimera plants were obtained from the endosperm of the seeds from inter-specific and ploidy crosses with tetraploid *Actinidia melanandra* [18]. In *Passiflora foetida*, a stable karyotype has been reported in endosperm-derived plants [47]. In the present study, flow cytometric analysis indicated that the variation in fluorescence intensity of the endosperm-derived plantlets was small, and karyotype analysis revealed that most of them had 2*n* = 3*x* = 24, suggesting that the ploidy level of the endosperm remained stable throughout the endosperm culture. Several approaches, such as flow cytometric analysis [48, 49], ITS region sequencing [50, 51], and karyotype analysis [48, 52] have been used to detect allotriploid plants. In the present study, flow cytometric analysis could not be used to distinguish them, ITS sequencing detected some of them, and karyotype analysis offered conclusive evidence that all investigated endosperm-derived plantlets were allotriploid plants. Thus, the confirmation methods commonly used in previous research on endosperm culture are also effective for allopolyploid plant production. However, more detailed investigations, such as chromosome observation with karyotype analysis, flow cytometric analysis with internal standards, and genetic analyses, are required. In addition to nuclear genome analyses, we sequenced genes in cytoplasmic genomes and found that cpDNA was maternally inherited in both embryos and endosperm-derived plantlets produced in the present study. It has been known that plastid genome is predominantly inherited through the maternal plant, and this seems to be the case for *Haemanthus* species. However, inheritance has been reported to be biparental and parental in plants, such as the genera *Actinidia* [53] and *Passiflora* [54]. Plastid genomes, which encode key genes for photosynthetic processes, including light reactions and carbon assimilation, are potential targets for plant breeding [55]. It is unknown whether the inheritance pattern is the same in embryo- and endosperm-derived plants; hence, further investigation is necessary for allotriploid production using endosperm cultures.

Further studies will focus on phenotypic and other characteristic analyses as the growth of the *Haemanthus* plants progresses. Plant regeneration from endosperm is often technically challenging [8], and the number of research on the morphological and agronomical characteristics is small. Mikovski et al. [47] compared the characteristics of the endosperm-derived triploid *Passiflora foetida* with those of diploids and found that the vegetative and floral structures of the endosperm-derived plants were all approximately larger than those of their diploid counterparts. It has been known that the alternation of ploidy levels and genome composition affects the phenotypes of plants produced naturally and artificially. In common wheat (*Triticum aestivum* subsp. *Aestivum*, AABBDD genome), it is thought to have emerged through the natural hybridization of *Triticum turgidum* L. (AABB genome) as the maternal progenitor and *Aegilops tauschii* Coss. (DD genome) as the paternal progenitor [56]. The genetic relationships of six *Brassica* species are described by the triangle of the U model [57] as follows: they share the same three core genomes of *B. rapa* (AA genome), *B. nigera* (BB genome), and *B. oleracea* (CC genome), and hybridization events result in the three allotetraploid species of *B. juncea* (AABB genome), *B. napus* (AACC genome), and *B. carinata* (BBCC genome). In *Lilium*, interspecific hybridization of *Lilium longiflorum* (LL genome) and Asiatic hybrids (AA genome) results in esthetically undesirable F_1_ hybrids (LA genome) with intermediate morphology between the parents; however, the allotriploid (LLA and ALA genome) backcrossed with F_1_ hybrids have been utilized as commercial cultivars [58]. Therefore, it is necessary to investigate the phenotypes of endosperm-derived allotriploid plantlets obtained in the present study and compare them with those of their parents and embryo-derived plantlets.

Allopolyploid plants contain both polyploidization and hybridization factors, and therefore, it is challenging to determine whether the desired traits are due to polyploidization, hybridization, or an interaction between them. Liqin et al. [59] compared the phenotypic traits and gene expression patterns of diploid parents, diploid full-sibs, allotriploid plants, and allotetraploid plants of *Populus* and reported that these different ploidy populations are good models for polyploidization and heterosis advantage studies. Another finding of the present study was the simultaneous production of diploid hybrids and allotriploid plantlets from the pairs of the embryo and endosperm in the same seeds, respectively. We also reported the production of embryo- and endosperm-derived *H*. *albiflos* plants at various ploidy levels previously [16, 24]. Therefore, it is suggested that the culture of each embryo and endosperm in the same seeds from self- and reciprocal crossing would result in the efficient production of plants, including homogenous embryo-derived-diploid and endosperm-derived triploid (autopolyploid) and heterogeneous embryo-derived diploid and endosperm-derived triploid (allotriploid) plants. This method is not limited to *Haemanthus* but has the potential to be applied to other plant species. It has been reported that the endosperm culture along with the embryos was more effective in certain plants, such as *Carica papaya* [14], *Morus alba* [13], and *Phlox drummondii* [15]. On the other hand, endosperm cultures were performed after removing the embryos to avoid contamination or unnecessary use, and these embryos were available but not for use in genera, such as *Actinidia* [12], *Lonicera* [17], and *Passiflora* [47]. The embryos of these plants could also be grown into plants, as in the present method, and they have the potential to be utilized as research materials for allopolyploid plant studies.

We believe that these findings will contribute to plant breeding and investigation on how the ploidy levels, parental genome composition, heterosis, and the direction of crossing influence morphological and agronomic characteristics.

## Conclusions

This study successfully produced allotriploid plants from the endosperm tissue of interspecific crosses between *H*. *pauculifolius* and *H*. *albiflos* using our previously established endosperm culture system. The endosperm tissues formed calli, which subsequently developed into plantlets. Flow cytometric analysis revealed that these plantlets retained their original ploidy levels. We conducted ITS sequencing and karyotype analysis to confirm the characteristics of endosperm-derived allotriploid plantlets with a parental chromosome ratio of 2:1 (maternal: paternal). To our knowledge, this is the first study to produce allotriploid hybrid plants. In addition, Sanger sequencing of cpDNA and mtDNA was conducted on both endosperm-derived and embryo-derived plants to investigate previously unknown patterns of cytoplasmic inheritance in endosperm-derived plants. The results revealed that cpDNA inheritance in endosperm-derived plants was exclusively maternal, consistent with that observed in embryo-derived plants. Furthermore, this study established a system for the simultaneous production of diploid and allotriploid hybrid plants from embryo and endosperm pairs in a single seed. These findings highlight the potential of endosperm culture as a novel and effective approach for producing allotriploid plants, while also providing valuable insights into polyploidization and hybridization.

## Supplementary Information

Additional file 1: Table S1. Selected sequences of the primer used in the present study based on Rønsted *et al*. (2012). Table S1 shows primer information and the related references used in this study.

Additional file 2: Table S2. Accession numbers of DNA sequences from previous studies. Table S2 indicates the accession numbers of DNA sequences from previous studies.

Additional file 3: Table S3. Callus formation and shoot and plantlet regeneration in the culture of the endosperm from the interspecific cross between *Haemanthuspauculifolius* and *H*. *albiflos. *Table S3 provides the detailed data of each callus line derived from embryos and endosperms after the interspecific cross between *Haemanthus pauculifolius* and *H*.*albiflos*.

Additional file 4: Table S4. The SNPs of ITS region in *Haemanthuspauculifolius*, *H*. *albiflos*, embryo-derived plantlets, and endosperm-derived plantlets from the same seeds. Table S4 shows SNPs of the ITS region in *Haemanthus* *pauculifolius*, *H*. *albiflos*, embryo-derived plantlets, and endosperm-derived plantlets from the same seeds.

Additional file 5: Table S5. Accession numbers obtained in the present study. Table S5 indicates accession numbers of DNA sequences registered in this study.

Additional file 6: Fig. S1. Electrophoresis images showing the amplification of the ITS region in *Haemanthus* *pauculifolius*,*H*. *albiflos*, and embryo- and endosperm-derived plantlets. Fig. S1 shows electrophoresis images showing the amplification of the ITS region in *Haemanthus pauculifolius*, *H*. *albiflos*, and embryo- and endosperm-derived plantlets.

Additional file 7: Fig. S2. Representative chromosome images and karyograms of 21 embryo-derived plantlets from each seed. Fig. S2 shows representative chromosome images and karyograms of 21 embryo-derived plantlets from each seed.

Additional file 8. Fig. S3. Representative chromosome images and karyograms of 16 endosperm-derived plantlets from each seed. Fig. S3 shows representative chromosome images and karyograms of 16 endosperm-derived plantlets from each seed.

## Data Availability

The sequence data have been registered and available in DDBJ with accession numbers LC851707 – LC851784. The accession numbers of each plant are listed in Table S5.

## Abbreviations

2,4-D: 2,4-Dichloro phenoxy acetic acid
cpDNA: Chloroplast DNA
BAP: 6-Benzylaminopurine
DAPI: 4′6-Diamino-2-phenylindole
ITS: Internal transcribed spacer
mtDNA: Mitochondrial DNA
MS: Murashige and Skoog
NAA: Potassium naphthalene-1-acetate
NCBI: National Center for Biotechnology Information
PCR: Polymerase chain reaction
PGRs: Plant growth regulators
Picloram: 4-Amino-3,5,6-trichloropicolinic acid
WAC: Weeks after culture

## References

[1] Iannicelli J, Guariniello J, Tossi VE, Regalado JJ, Di Ciaccio L, van Baren CM, Pitta Álvarez SI, Escandón AS. The “polyploid effect” in the breeding of aromatic and medicinal species. Sci Hortic (Amsterdam). 2020;260:108854.

[2] Stebbins GL Jr. Types of polyploids; their classification and significance. Adv Genet. 1947;1:403–29. 20259289 10.1016/s0065-2660(08)60490-3

[3] Comai L. The advantages and disadvantages of being polyploid. Nat Rev Genet. 2005;6:836–46. 16304599 10.1038/nrg1711

[4] Sattler MC, Carvalho CR, Clarindo WR. The polyploidy and its key role in plant breeding. Planta. 2016;243:281–96. 26715561 10.1007/s00425-015-2450-x

[5] Thomas TD, Chaturvedi R. Endosperm culture: a novel method for triploid plant production. Plant Cell Tissue Organ Cult. 2008;93:1–14.

[6] Eng WH, Ho WS. Polyploidization using colchicine in horticultural plants: a review. Sci Hortic (Amsterdam). 2019;246:604–17.

[7] Niazian M, Nalousi AM. Artificial polyploidy induction for improvement of ornamental and medicinal plants. Plant Cell Tissue Organ Cult. 2020;142:447–69.

[8] Wang X, Cheng ZM, Zhi S, Xu F. Breeding triploid plants: a review. Czech J Genet Plant Breed. 2016;52:41–54.

[9] Williams JH, Friedman WE. Identification of diploid endosperm in an early angiosperm lineage. Nature. 2002;415:522–6. 11823859 10.1038/415522a

[10] Ikeuchi M, Ogawa Y, Iwase A, Sugimoto K. Plant regeneration: cellular origins and molecular mechanisms. Development. 2016;143:1442–51. 27143753 10.1242/dev.134668

[11] Gmitter FG, Ling XB, Deng XX. Induction of triploid *Citrus* plants from endosperm calli in vitro. Theor Appl Genet. 1990;80:785–90. 24221109 10.1007/BF00224192

[12] Asakura I, Hoshino Y. Endosperm-derived triploid plant regeneration in diploid *Actinidia**kolomikta*, a cold-hardy kiwifruit relative. Sci Hortic (Amsterdam). 2017;219:53–9.

[13] Thomas TD, Bhatnagar AK, Bhojwani SS. Production of triploid plants of mulberry (*Morus**alba* L) by endosperm culture. Plant Cell Rep. 2000;19:395–9. 30754793 10.1007/s002990050746

[14] Sun DQ, Lu XH, Liang GL, Guo QG, Mo YW, Xie JH. Production of triploid plants of papaya by endosperm culture. Plant Cell Tissue Organ Cult. 2011;104:23–9.

[15] Razdan Tiku A, Razdan MK, Raina SN. Production of triploid plants from endosperm cultures of *Phlox**drummondii*. Biol Plant. 2014;58:153–8.

[16] Nakano A, Mii M, Hoshino Y. Simultaneous production of triploid and hexaploid plants by endosperm culture with colchicine treatment in diploid *Haemanthus**albiflos*. Plant Cell Tissue Organ Cult. 2021;144:661–9.

[17] Miyashita T, Ohashi T, Shibata F, Araki H, Hoshino Y. Plant regeneration with maintenance of the endosperm ploidy level by endosperm culture in *Lonicera**caerulea* var. *emphyllocalyx*. Plant Cell Tissue Organ Cult. 2009;98:291–301.

[18] Kin MS, Fraser LG, Harvey CF. Initiation of callus and regeneration of plantlets from endosperm of *Actinidia* interspecific hybrids. Sci Hortic (Amsterdam). 1990;44:107–17.

[19] Wang CC, Lu WL, Kuang BJ. Study on the hybrid endosperm culture of wheat-rye in vitro. J Integr Plant Biol. 1982;24:420–6. (in Chinese).

[20] Duncan G, Joubert E. 1023 *Haemanthus**humanii*: Amaryllidaceae. Curtis’s Bot Mag. 2022;39:279–94.

[21] Snijman DA. A revision of the genus *Haemanthus* L. (Amaryllidaceae). J S Afr Bot Suppl. 1984;12:1–139.

[22] Snijman DA, van Wyk AE. A new species of *Haemanthus* (Amaryllidaceae) from the Eastern Transvaal escarpment. South Africa S Afr J Bot. 1993;59:247–50.

[23] Satô D. Karyotype alteration and phylogeny. IV: Karyotypes in Amaryllidaceae with special reference to the SAT-chromosome. Cytologia. 1938;9:203–42.

[24] Nakano A, Hoshino Y. Production of tetraploid and octoploid *Haemanthus**albiflos* plants using immature embryo-derived embryogenic calli treated with colchicine. Plant Cell Tissue Organ Cult. 2022;149:747–52.

[25] Murashige T, Skoog F. A revised medium for rapid growth and bio assays with tobacco tissue cultures. Physiol Plant. 1962;15:473–97.

[26] Mishiba KI, Ando T, Mii M, Watanabe H, Kokubun H, Hashimoto G, Marchesi E. Nuclear DNA content as an index character discriminating taxa in the genus *Petunia**sensu* Jussieu (Solanaceae). Ann Bot. 2000;85:665–73.

[27] White TJ, Bruns T, Lee S, Taylor J. Amplification and direct sequencing of fungal ribosomal RNA genes for phylogenetics. In: Innis MA, Gelfand DH, Sninsky JJ, White TJ, editors. PCR protocols. San Diego, CA: Academic Press; 1990. p. 315–22.

[28] Rønsted N, Symonds MRE, Birkholm T, Christensen SB, Meerow AW, Molander M, Mølgaard P, Petersen G, Rasmussen N, van Staden J, Stafford GI, Jäger AK. Can phylogeny predict chemical diversity and potential medicinal activity of plants? A case study of Amaryllidaceae. BMC Evol Biol. 2012;12:182. 22978363 10.1186/1471-2148-12-182 PMC3499480

[29] Tanaka R, Taniguchi K. A banding method for plant chromosomes. Jpn J Genet. 1975;50:163–7.

[30] Fukui K, Iijima K. Somatic chromosome map of rice by imaging methods. Theor Appl Genet. 1991;81:589–96. 24221372 10.1007/BF00226723

[31] Kores PJ, Weston PH, Molvray M, Chase MW. Phylogenetic relationships within the Diurideae (Orhidaceae): inferences from plastid *matK* DNA sequences. In: Wilson KL, Morrison DA, editors. Monocots. Systematics & evolution. Collingwood, Victoria: CSIRO Publishing; 2000. p. 449–56.

[32] Johnson LA, Soltis DE. *matK* DNA sequences and phylogenetic reconstruction in Saxifragaceae s. str. Syst Bot. 1994;19:143–56.

[33] Taberlet P, Gielly L, Pautou G, Bouvet J. Universal primers for amplification of three non-coding regions of chloroplast DNA. Plant Mol Biol. 1991;17:1105–9. 1932684 10.1007/BF00037152

[34] Demesure B, Sodzi N, Petit RJ. A set of universal primers for amplification of polymorphic non-coding regions of mitochondrial and chloroplast DNA in plants. Mol Ecol. 1995;4:129–31. 7711952 10.1111/j.1365-294x.1995.tb00201.x

[35] Larsen MM, Adsersen A, Davis AP, Lledó MD, Jäger AK, Rønsted N. Using a phylogenetic approach to selection of target plants in drug discovery of acetylcholinesterase inhibiting alkaloids in Amaryllidaceae tribe Galantheae. Biochem Syst Ecol. 2010;38:1026–34.

[36] Sun H, McLewin W, Fay MF. Molecular phylogeny of *Helleborus* (*Ranunculaceae*), with an emphasis on the East Asian-Mediterranean disjunction. Taxon. 2001;50:1001–18.

[37] Cuenca A, Petersen G, Seberg O, Jahren AH. Genes and processed paralogs co-exist in plant mitochondria. J Mol Evol. 2012;74:158–69. 22484699 10.1007/s00239-012-9496-1

[38] Guzzo F, Ceoldo S, Andreetta F, Levi M. In vitro culture from mature seeds of *Passiflora* species. Sci Agric. 2004;61:108–13.

[39] James DJ, Passey AJ, Charles DD. Adventitious embryogenesis and the in vitro culture of apple seed parts. J Plant Physiol. 1984;115:217–29. 23194578 10.1016/S0176-1617(84)80124-8

[40] Long Y, Yang Y, Pan G, Shen Y. New insights into tissue culture plant-regeneration mechanisms. Front Plant Sci. 2022;13:926752. 35845646 10.3389/fpls.2022.926752 PMC9280033

[41] Hoshino Y, Miyashita T, Thomas TD. In vitro culture of endosperm and its application in plant breeding: approaches to polyploidy breeding. Sci Hortic (Amsterdam). 2011;130:1–8.

[42] Scott RJ, Spielman M, Bailey J, Dickinson HG. Parent-of-origin effects on seed development in *Arabidopsis**thaliana*. Development. 1998;125:3329–41. 9693137 10.1242/dev.125.17.3329

[43] Bushell C, Spielman M, Scott RJ. The basis of natural and artificial postzygotic hybridization barriers in *Arabidopsis* species. Plant Cell. 2003;15:1430–42. 12782734 10.1105/tpc.010496 PMC156377

[44] Ishikawa R, Ohnishi T, Kinoshita Y, Eiguchi M, Kurata N, Kinoshita T. Rice interspecies hybrids show precocious or delayed developmental transitions in the endosperm without change to the rate of syncytial nuclear division. Plant J. 2011;65:798–806. 21251103 10.1111/j.1365-313X.2010.04466.x

[45] Sekine D, Ohnishi T, Furuumi H, Ono A, Yamada T, Kurata N, Kinoshita T. Dissection of two major components of the post-zygotic hybridization barrier in rice endosperm. Plant J. 2013;76:792–9. 24286595 10.1111/tpj.12333

[46] Gui Y, Hong S, Ke S, Skirvin RM. Fruit and vegetative characteristics of endosperm-derived kiwifruit (*Actinidia**chinensis* F) plants. Euphytica. 1993;71:57–62.

[47] Mikovski AI, da Silva NT, Silva LAS, Machado M, de Souza Barbosa LC, Reis AC, et al. From endosperm to triploid plants: a stepwise characterization of the de novo shoot organogenesis and morpho-agronomic aspects of an ornamental passion fruit (*Passiflora**foetida* L.). Plant Cell Tissue Organ Cult. 2021;147:239–53.

[48] Hang TTM, Shigyo M, Yamauchi N, Tashiro Y. Production and characterization of alien chromosome additions in shallot (*Allium**cepa* L. Aggregatum group) carrying extra chromosome(s) of Japanese bunching onion (*A*. *fistulosum* L.). Genes Genet Syst. 2004;79:263–9. 10.1266/ggs.79.263 15599056

[49] Bennert HW, Horn K, Kauth M, Fuchs J, Jakobsen ISB, Øllgaard B, Schnittler M, Steinberg M, Viane R. Flow cytometry confirms reticulate evolution and reveals triploidy in Central European *Diphasiastrum* taxa (Lycopodiaceae, Lycophyta). Ann Bot. 2011;108:867–76. 21835817 10.1093/aob/mcr208 PMC3177684

[50] Hodkinson TR, Chase MW, Takahashi C, Leitch IJ, Bennett MD, Renvoize SA. The use of DNA sequencing (ITS and *trnL-F*), AFLP, and fluorescent in situ hybridization to study allopolyploid *Miscanthus* (Poaceae). Am J Bot. 2002;89:279–86. 21669737 10.3732/ajb.89.2.279

[51] Kaplan Z, Fehrer J. Molecular evidence for a natural primary triple hybrid in plants revealed from direct sequencing. Ann Bot. 2007;99:1213–22. 17478544 10.1093/aob/mcm072 PMC3243585

[52] Schoenmakers HCH, Wolters AMA, Nobel EM, De Klein CMJ, Koornneef M. Allotriploid somatic hybrids of diploid tomato (*Lycopersicon**esculentum* Mill.) and monoploid potato (*Solanum**tuberosum* L.). Theor Appl Genet. 1993;87:328–36. 10.1007/BF01184919 24190258

[53] Li D, Qi X, Li X, Li L, Zhong C, Huang H. Maternal inheritance of mitochondrial genomes and complex inheritance of chloroplast genomes in *Actinidia* Lind.: evidences from interspecific crosses. Mol Genet Genomics. 2013;288:101–10. 10.1007/s00438-012-0732-6 23337924

[54] Hansen AK, Escobar LK, Gilbert LE, Jansen RK. Paternal, maternal, and biparental inheritance of the chloroplast genome in *Passiflora* (Passifloraceae): implications for phylogenetic studies. Am J Bot. 2007;94:42–6. 21642206 10.3732/ajb.94.1.42

[55] Nakazato I, Okuno M, Yamamoto H, Tamura Y, Itoh T, Shikanai T, Takanashi H, Tsutsumi N, Arimura SI. Targeted base editing in the plastid genome of *Arabidopsis**thaliana*. Nat Plants. 2021;7:906–13. 34211131 10.1038/s41477-021-00954-6 PMC8289735

[56] Matsuoka Y, Mori N. Reproductive and genetic roles of the maternal progenitor in the origin of common wheat (*Triticum**aestivum* L.). Ecol Evol. 2020;10:13926–37. 10.1002/ece3.6985 PMC7771132 33391691

[57] Nagaharu U. Genome analysis in *Brassica* with special reference to the experimental formation of *B*. *napus* and peculiar mode of fertilization. J Jpn Bot. 1935;7:389–452.

[58] Marasek-Ciolakowska A, Nishikawa T, Shea DJ, Okazaki K. Breeding of lilies and tulips-interspecific hybridization and genetic background. Breed Sci. 2018;68:35–52. 29681746 10.1270/jsbbs.17097 PMC5903980

[59] Liqin G, Jianguo Z, Xiaoxia L, Guodong R. Polyploidy-related differential gene expression between diploid and synthesized allotriploid and allotetraploid hybrids of *Populus*. Mol Breed. 2019;39:1–15.

