# Supplementary material for: Endosperm culture-based allotriploid hybrid production from an interspecific cross of Haemanthus spp.: new insights into polyploidization and hybridization
Source: BMC Plant Biol. 2025 Feb 6;25:158. doi: 10.1186/s12870-025-06181-x (PMC11800442; doi:10.1186/s12870-025-06181-x)
Supplement: Supplementary file 1 — Additional file 1: Table S1. Selected sequences of the primer used in the present study based on Rønsted et al. (2012). Table S1 shows primer information and the related references used in this study. [file 12870_2025_6181_MOESM1_ESM.pdf]

Table S1.

Selected sequences of the primer used in this study based on [28] Rønsted *et al*. (2012).

Primer name	Target	Sequence (5’–3’)	Direction	Reference
ITS5	ITS region	GGA AGT AAA AGT CGT AAC AAG G	Forward	[27]
ITS4	ITS region	TCC TCC GCT TAT TGA TAT GC	Reverse	[27]
trnC	*trnL-trnF* region	CGA AAT CGG TAG ACG CTA CG	Forward	[33]
trnF	*trnL-trnF* region	ATT TGA ACT GGT GAC ACG AG	Reverse	[33]
19F	*matK* gene	CGT TCT GAC CAT ATT GCA CTA TG	Forward	[31]
2R	*matK* gene	AAC TAG TCG GAT GGA GTA G	Reverse	[32]
KatF*	*matK* gene	ATC GGG CCA TCC TAT TAG	Forward	[35]
1326R*	*matK* gene	TCT AGC ACA CGA AAG TCG AAG T	Reverse	[36]
nad1eB	*nad1* gene	GCA TTA CGA TCT GCA GCT CA	Forward	[34]
nad1eCR	*nad1* gene	GGA GCT CGA TTA GTT TCT GC	Reverse	[34]
nad1iB2*	*nad1* gene	GTC GAG CAT ACG ACG ATG CCG C	Forward	[37]
nad1iB2R*	*nad1* gene	GGC GGC ATC GTC GTA TGC TCG	Reverse	[37]

* Primers used for Sanger sequencing.

References

[27] White TJ, Bruns T, Lee S, Taylor J. Amplification and direct sequencing of fungal ribosomal RNA genes for phylogenetics. In: Innis MA, Gelfand DH, Sninsky JJ, White TJ, ed. PCR protocols. San Diego, CA, USA: Academic Press, 1990; 315-322.

[28] Rønsted N, Symonds MRE, Birkholm T, Christensen SB, Meerow AW, Molander M, Mølgaard P, Petersen G, Rasmussen N, van Staden J, Stafford GI, Jäger AK. Can phylogeny predict chemical diversity and potential medicinal activity of plants? A case study of Amaryllidaceae. BMC Evol. Biol. 2012; 12: 182.

[31] Kores PJ, Weston PH, Molvray M, Chase MW. Phylogenetic relationships within the Diurideae (Orhidaceae): inferences from plastid *matK* DNA sequences. In: Wilson KL, Morrison DA, ed. Monocots. Systematics & evolution. Collingwood, Victoria, Australia: CSIRO Publishing, 2000; 449-456.

[32] Johnson LA, Soltis DE. *matK* DNA sequences and phylogenetic reconstruction in Saxifragaceae s. str. Syst. Bot. 1994; 19: 143-156.

[33] Taberlet P, Gielly L, Pautou G, Bouvet J. Universal primers for amplification of three non-coding regions of chloroplast DNA. Plant Mol. Biol. 1991; 17: 1105-1109.

[34] Demesure B, Sodzi N, Petit RJ. A set of universal primers for amplification of polymorphic non-coding regions of mitochondrial and chloroplast DNA in plants. Mol. Ecol. 1995; 4: 129-131.

[35] Larsen MM, Adsersen A, Davis AP, Lledó MD, Jäger AK, Rønsted N. Using a phylogenetic approach to selection of target plants in drug discovery of acetylcholinesterase inhibiting alkaloids in Amaryllidaceae tribe Galantheae. Biochem. Syst. Ecol. 2010; 38: 1026-1034.

[36] Sun H, McLewin W, Fay MF. Molecular phylogeny of *Helleborus* (*Ranunculaceae*), with an emphasis on the East Asian-Mediterranean disjunction. Taxon 2001; 50: 1001-1018.

[37] Cuenca A, Petersen G, Seberg O, Jahren AH. Genes and processed paralogs co-exist in plant mitochondria. J. Mol. Evol. 2012; 74: 158-169.
