# Supplementary material for: Endosperm culture-based allotriploid hybrid production from an interspecific cross of Haemanthus spp.: new insights into polyploidization and hybridization
Source: BMC Plant Biol. 2025 Feb 6;25:158. doi: 10.1186/s12870-025-06181-x (PMC11800442; doi:10.1186/s12870-025-06181-x)
Supplement: Supplementary file 2 — Additional file 2: Table S2. Accession numbers of DNA sequences from previous studies. Table S2 indicates the accession numbers of DNA sequences from previous studies. [file 12870_2025_6181_MOESM2_ESM.pdf]

Table S2
Accession numbers of DNA sequences from previous studies.

NCBI accession number	Target	Species
AY280356	ITS region	*Haemanthus albiflos*
HM140803	ITS region	*Haemanthus albiflos*
HM140808	ITS region	*Haemanthus humilis* subsp. *hirsutus*
HM140811	ITS region	*Haemanthus sanguineus*
JX464554	*matK* gene	*Haemanthus amarylloides*
JX464555	*matK* gene	*Haemanthus coccineus*
JX903563	*matK* gene	*Haemanthus albiflos*
AY278966	*trnL-trnF* region	*Haemanthus albiflos*
AY278980	*trnL-trnF* region	*Haemanthus albiflos*
EU523831	*trnL-trnF* region	*Haemanthus albiflos*
EU523832	*trnL-trnF* region	*Haemanthus albiflos*
EU523839	*trnL-trnF* region	*Haemanthus pauculifolius*
JX464449	*nad1* gene	*Haemanthus amarylloides*
JX464450	*nad1* gene	*Haemanthus coccineus*
JX464451	*nad1* gene	*Haemanthus humilis* subsp. *hirsutus*
JX464452	*nad1* gene	*Haemanthus montanus*
JX464453	*nad1* gene	*Haemanthus sanguineus*
