# Supplementary material for: Endosperm culture-based allotriploid hybrid production from an interspecific cross of Haemanthus spp.: new insights into polyploidization and hybridization
Source: BMC Plant Biol. 2025 Feb 6;25:158. doi: 10.1186/s12870-025-06181-x (PMC11800442; doi:10.1186/s12870-025-06181-x)
Supplement: Supplementary file 3 — Additional file 3: Table S3. Callus formation and shoot and plantlet regeneration in the culture of the endosperm from the interspecific cross between Haemanthuspauculifolius and H. albiflos. Table S3 provides the detailed data of each callus line derived from embryos and endosperms after the interspecific cross between Haemanthus pauculifolius and H.albiflos. [file 12870_2025_6181_MOESM3_ESM.pdf]

Table S3

Callus formation and shoot and plantlet regeneration in the culture of the endosperm from the interspecific cross between *Haemanthus pauculifolius* and *H. albiflos*.

Sample ID	CIM: MS supplemented with 5 mg L^{-1} Picloram + 5 mg L^{-1} BAP									CIM: MS supplemented with 5 mg L^{-1} 2,4-D + 5 mg L^{-1} BAP									Endosperm produced plantlet
	No. of explants	Callus induction			Shoot regeneration on the SIM					No. of explants	Callus induction			Shoot regeneration on the SIM					
		4WAC	8WAC	Last	Callus	1/2MS	MS	MS + PGR	Shoot		4WAC	8WAC	Last	Callus	1/2MS	MS	MS + PGR	Shoot	
EN1-1	4	0	4	4	✓	✓	✓		✓	4	0	3	3	✓					✓
EN1-2	4	0	4	4	✓			✓	✓	4	0	1	1	✓					✓
EN2-1	4	0	3	3	✓	✓	✓	✓	✓	4	0	0	0						✓
EN2-2	4	0	2	2	✓	✓		✓	✓	4	0	0	4	✓					✓
EN2-3	3	0	0	0						4	0	0	0						
EN3-1	4	0	2	2	✓	✓		✓	✓	4	1	2	4	✓					✓
EN3-2	4	0	4	4	✓	✓		✓	✓	4	0	4	4	✓	✓		✓	✓	✓
EN4-1	4	2	3	4	✓	✓	✓	✓	✓	4	0	4	4	✓	✓	✓	✓	✓	✓
EN4-2	4	2	2	2	✓		✓	✓	✓	4	1	2	2	✓					✓
EN5-1	4	3	4	4	✓	✓	✓	✓	✓	4	0	3	4	✓	✓	✓	✓	✓	✓
EN6-1	4	1	2	4	✓		✓	✓	✓	4	3	0	0						✓
EN7-1	4	2	4	4	✓			✓	✓	4	0	4	4	✓	✓	✓	✓	✓	✓
EN7-2	4	0	2	4	✓		✓	✓	✓	4	2	0	0						✓
EN8-1	4	2	3	4	✓	✓	✓	✓	✓	4	0	0	0						✓
EN9-1	4	0	2	2	✓	✓	✓	✓	✓	4	0	0	1	✓					✓
EN9-2	4	0	4	4	✓	✓	✓	✓	✓	4	0	0	4	✓					✓
EN10-1	4	0	0	0						4	0	0	0						
EN10-2	3	0	0	0						3	0	0	0						
EN10-3	4	0	0	0						4	0	0	0						
EN11-1	4	1	4	4	✓	✓	✓	✓	✓	4	0	4	4	✓		✓		✓	✓
EN11-2	4	0	4	4	✓		✓	✓	✓	4	0	1	4	✓	✓	✓	✓	✓	✓
EN12-1	4	3	3	4	✓	✓	✓	✓	✓	4	3	3	3	✓	✓	✓	✓	✓	✓
22	86	16	56	63	18	12	13	17	18	87	10	31	46	14	6	6	6	7	18

CIM: Callus induction medium, SIM: Shoot induction medium, PGR: Plant growth regulators (NAA + BAP), WAC: weeks after culture.
