# Supplementary material for: Endosperm culture-based allotriploid hybrid production from an interspecific cross of Haemanthus spp.: new insights into polyploidization and hybridization
Source: BMC Plant Biol. 2025 Feb 6;25:158. doi: 10.1186/s12870-025-06181-x (PMC11800442; doi:10.1186/s12870-025-06181-x)
Supplement: Supplementary file 4 — Additional file 4: Table S4. The SNPs of ITS region in Haemanthuspauculifolius, H. albiflos, embryo-derived plantlets, and endosperm-derived plantlets from the same seeds. Table S4 shows SNPs of the ITS region in Haemanthus pauculifolius, H. albiflos, embryo-derived plantlets, and endosperm-derived plantlets from the same seeds. [file 12870_2025_6181_MOESM4_ESM.pdf]

Table S4

The SNPs of ITS region in *Haemanthus pauculifolius*, *H. albiflos*, embryo-derived plantlets, and endosperm-derived plantlets from the same seeds.

Embryo-derived plantlet (EM)						Endosperm-derived plantlet (EN)						
	ITS1		5.8S rRNA				ITS1		5.8S rRNA			
Sample ID	ITS5	ITS4	ITS5	ITS4	Hybrid	Sample ID	ITS5	ITS4	ITS5	ITS4	Hybrid	Hybrid seed
H. pauculifolius	Y	Y	G	G		*H. pauculifolius*	Y	Y	G	G		
H. albiflos	C	C	R	R		*H. albiflos*	C	C	R	R		
EM1-1	Y	Y	R	R	✓	EN1-1	Y	Y	G	G		✓
EM1-2	Y	Y	G	G								
EM2-1	Y	Y	G	G		EN2-1	Y	Y	G	G		
EM2-2	Y	Y	G	G		EN2-2	Y	Y	G	G		
EM2-3	Y	Y	G	G								
EM3-1	Y	Y	R	R	✓	EN3-1	Y	Y	G	R	✓	✓
EM3-2	Y	Y	R	R	✓	EN3-2	Y	Y	R	R	✓	✓
EM4-1	Y	Y	R	R	✓	EN4-1	Y	Y	G	G		✓
EM4-2	Y	Y	G	G		EN4-2	Y	Y	G	G		
EM5-1	Y	Y	G	G		EN5-1	Y	Y	G	G		
EM6-1	Y	Y	R	R	✓	EN6-1	Y	Y	G	R	✓	✓
EM7-1	Y	Y	G	G		EN7-1	Y	Y	G	G		
EM7-2	Y	Y	G	G		EN7-2	Y	Y	G	G		
EM8-1	Y	Y	R	R	✓	EN8-1	Y	Y	G	R	✓	✓
EM9-1	Y	Y	G	G		EN9-1	Y	Y	G	G		
EM9-2	Y	Y	G	G		EN9-2	Y	Y	G	G		
EM10-1	Y	Y	R	R	✓							✓
EM10-2	Y	Y	G	G								
EM11-1	Y	Y	G	G		EN11-1	Y	Y	G	G		
EM11-2	Y	Y	R	R	✓							✓
EM12-1	Y	Y	G	G		EN12-1	Y	Y	G	G		
Total					8						4	8

“Y” (T + C) and “R” (A + G) mean that one-base substitutions in the same individual were detected from two overlapping peaks.
