# Supplementary material for: Endosperm culture-based allotriploid hybrid production from an interspecific cross of Haemanthus spp.: new insights into polyploidization and hybridization
Source: BMC Plant Biol. 2025 Feb 6;25:158. doi: 10.1186/s12870-025-06181-x (PMC11800442; doi:10.1186/s12870-025-06181-x)
Supplement: Supplementary file 5 — Additional file 5: Table S5. Accession numbers obtained in the present study. Table S5 indicates accession numbers of DNA sequences registered in this study. [file 12870_2025_6181_MOESM5_ESM.pdf]

# ITS (Embryo-derived plantlets)

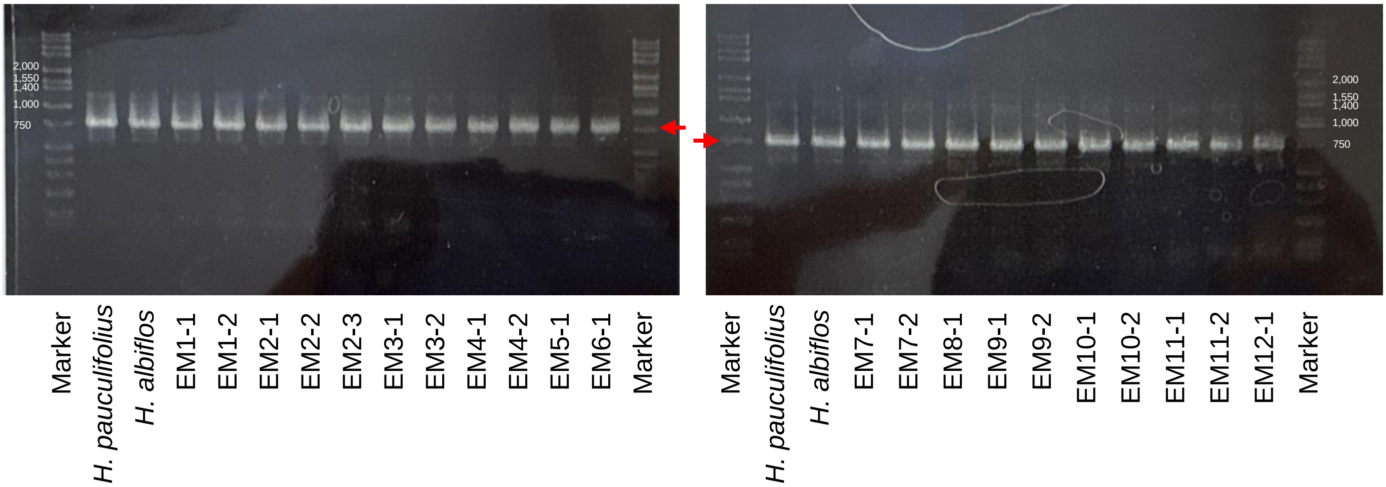


# ITS (Endosperm-derived plantlets)

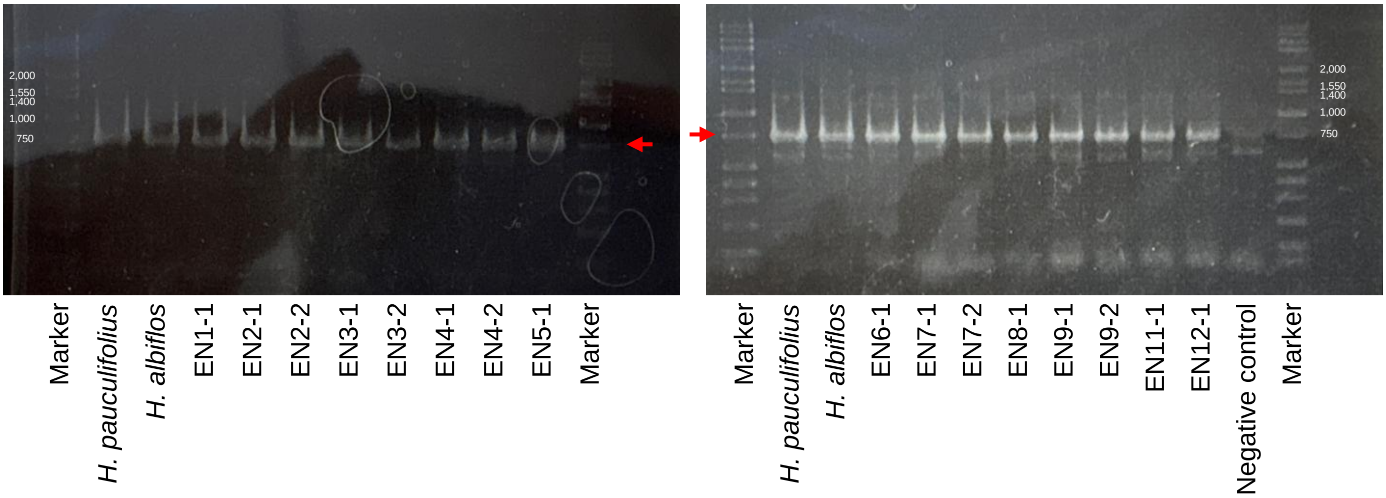


Fig. S1

Electrophoresis images showing the amplification of the ITS region in *Haemanthus pauculifolius*, *H. albiflos*, and embryo- and endosperm-derived plantlets.

The ITS regions were amplified using the forward primer ITS5 and the reverse primer ITS4. The marker used was the Wide-Range DNA Ladder (50–10,000 bp; Takara Bio, Shiga, Japan). Arrows indicate the target DNA bands.
