# Supplementary material for: Endosperm culture-based allotriploid hybrid production from an interspecific cross of Haemanthus spp.: new insights into polyploidization and hybridization
Source: BMC Plant Biol. 2025 Feb 6;25:158. doi: 10.1186/s12870-025-06181-x (PMC11800442; doi:10.1186/s12870-025-06181-x)
Supplement: Supplementary file 6 — Additional file 6: Fig. S1. Electrophoresis images showing the amplification of the ITS region in Haemanthus pauculifolius,H. albiflos, and embryo- and endosperm-derived plantlets. Fig. S1 shows electrophoresis images showing the amplification of the ITS region in Haemanthus pauculifolius, H. albiflos, and embryo- and endosperm-derived plantlets. [file 12870_2025_6181_MOESM6_ESM.pdf]

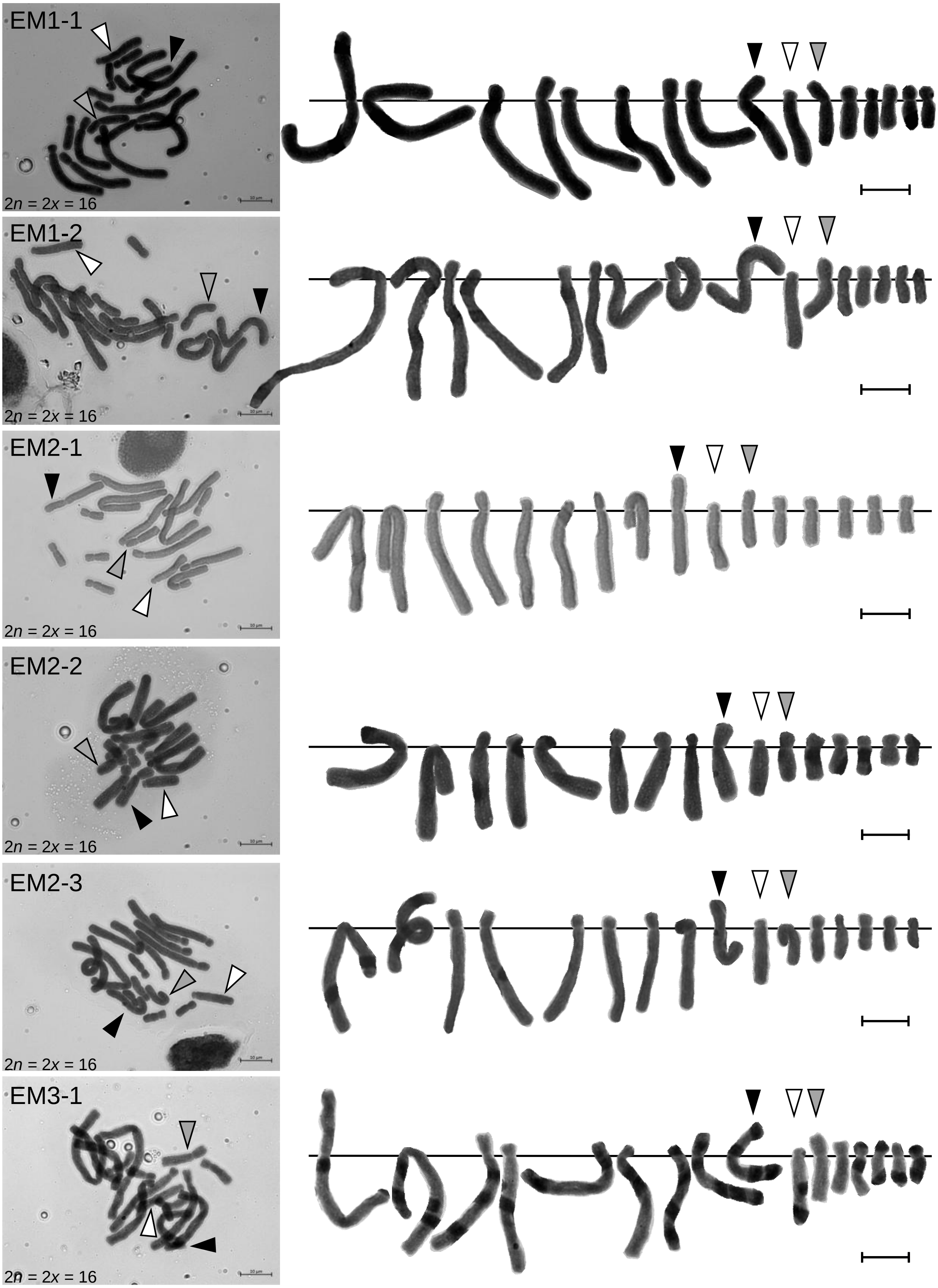

Fig. S2

Representative chromosome images and karyograms of 21 embryo-derived plantlets from each seed. Each arrowhead means the characteristic chromosome of *H*. *pauculifolius* (black) and *H*. *albiflos* (white and gray). Scale bars = 10 µm.

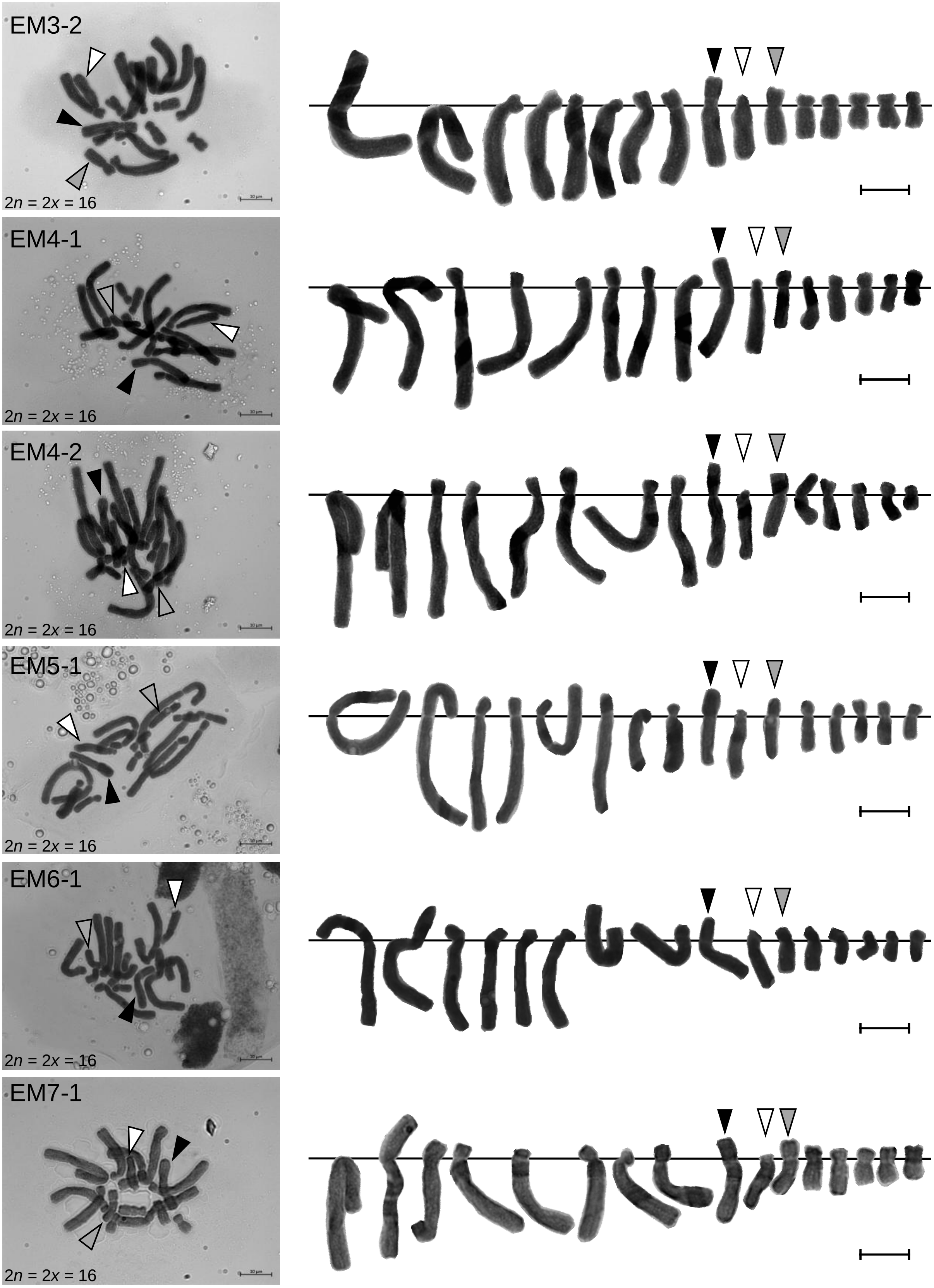

Fig. S2 Cont.
Representative chromosome images and karyograms of 21 embryo-derived plantlets from each seed. Each arrowhead means the characteristic chromosome of *H. pauculifolius* (black) and *H. albiflos* (white and gray). Scale bars = 10 µm.

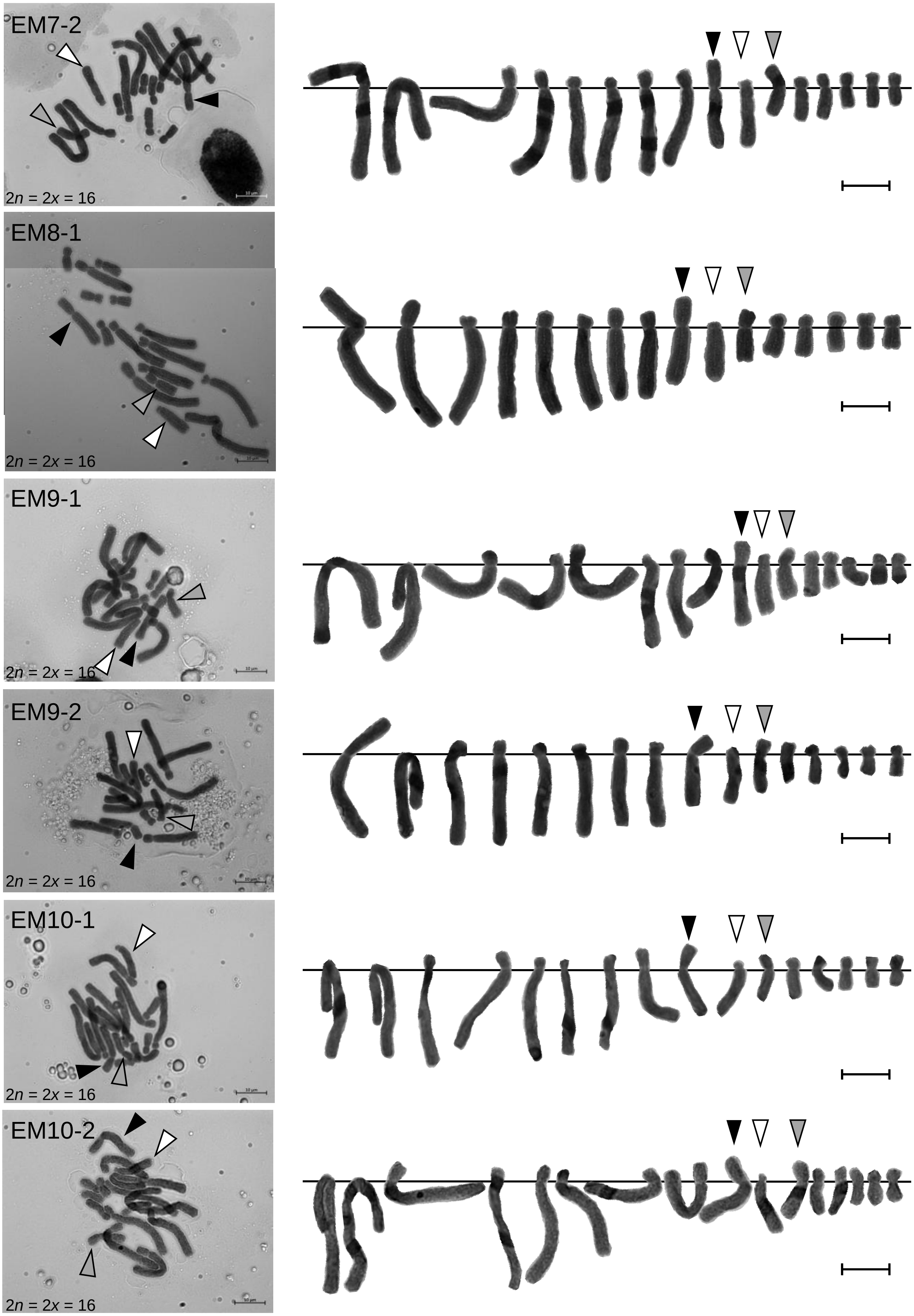

Fig. S1 Cont.
Representative chromosome images and karyograms of 21 embryo-derived plantlets from each seed. Each arrowhead means the characteristic chromosome of *H*. *pauculifolius* (black) and *H*. *albiflos* (white and gray). Scale bars = 10 µm.

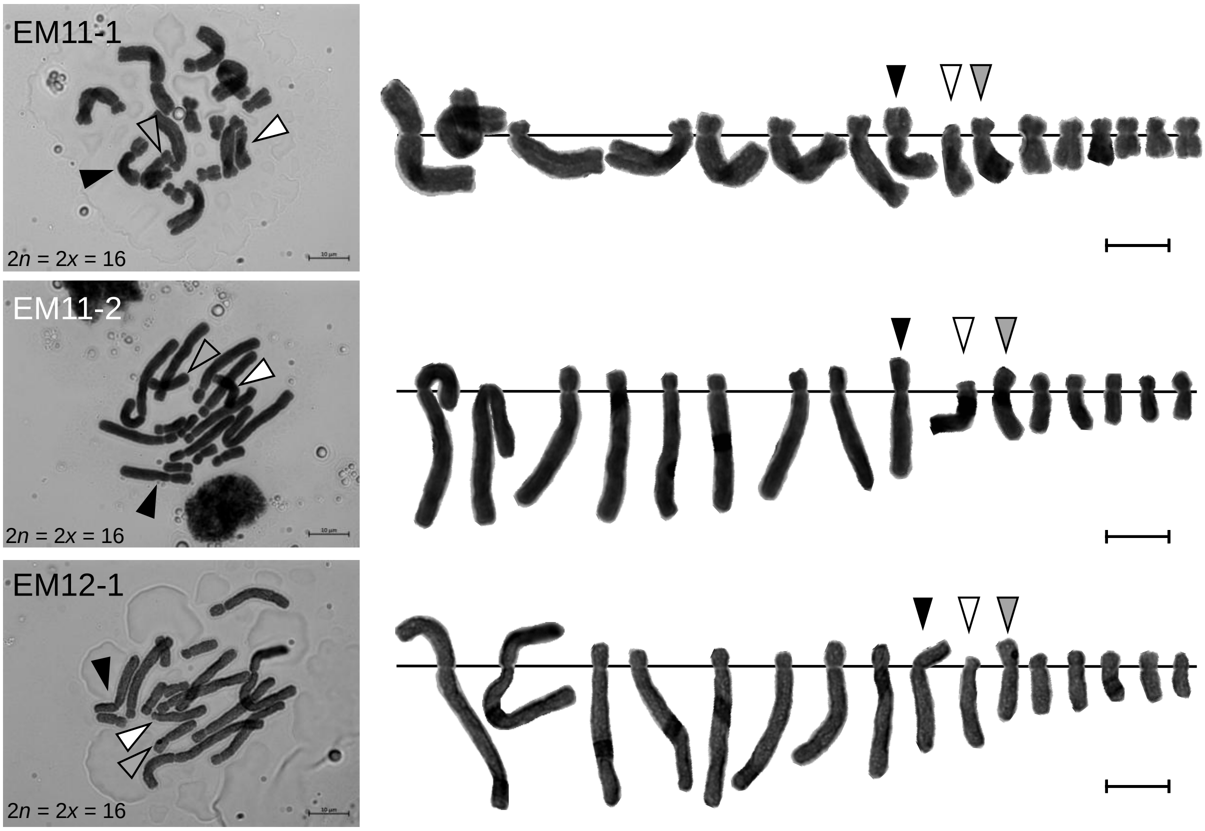

Fig. S2 Cont.
Representative chromosome images and karyograms of 21 embryo-derived plantlets from each seed. Each arrowhead means the characteristic chromosome of *H*. *pauculifolius* (black) and *H*. *albiflos* (white and gray). Scale bars = 10 µm.
