# Supplementary material for: Endosperm culture-based allotriploid hybrid production from an interspecific cross of Haemanthus spp.: new insights into polyploidization and hybridization
Source: BMC Plant Biol. 2025 Feb 6;25:158. doi: 10.1186/s12870-025-06181-x (PMC11800442; doi:10.1186/s12870-025-06181-x)
Supplement: Supplementary file 7 — Additional file 7: Fig. S2. Representative chromosome images and karyograms of 21 embryo-derived plantlets from each seed. Fig. S2 shows representative chromosome images and karyograms of 21 embryo-derived plantlets from each seed. [file 12870_2025_6181_MOESM7_ESM.pdf]

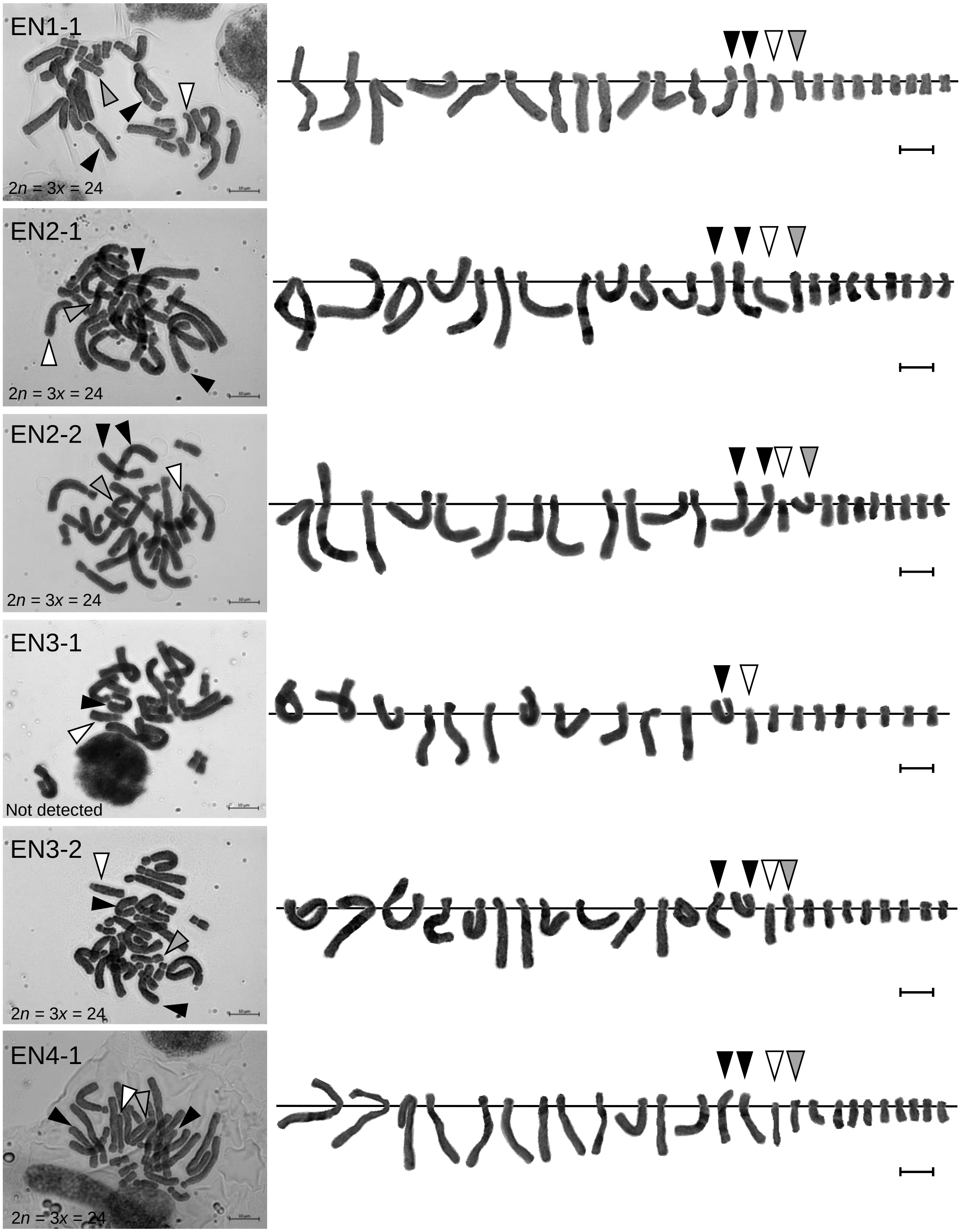


Fig. S3

Representative chromosome images and karyograms of 16 endosperm-derived plantlets from each seed. Each arrowhead means the characteristic chromosome of *H. pauculifolius* (black) and *H. albiflos* (white and gray). Scale bars = 10 µm.

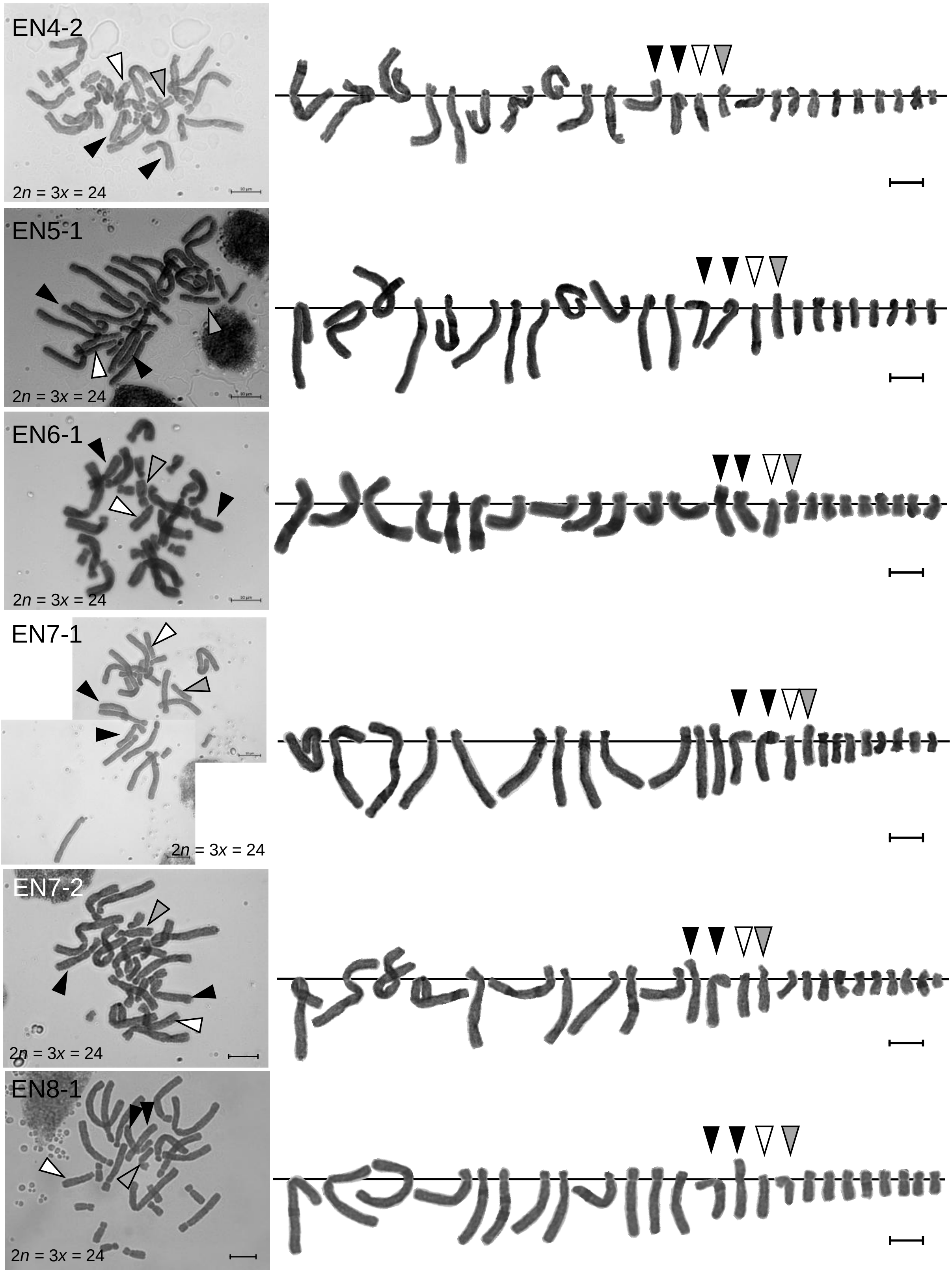


Fig. S3 Cont.
Representative chromosome images and karyograms of 16 endosperm-derived plantlets from each seed. Each arrowhead means the characteristic chromosome of *H*. *pauculifolius* (black) and *H*. *albiflos* (white and gray). Scale bars = 10 µm.

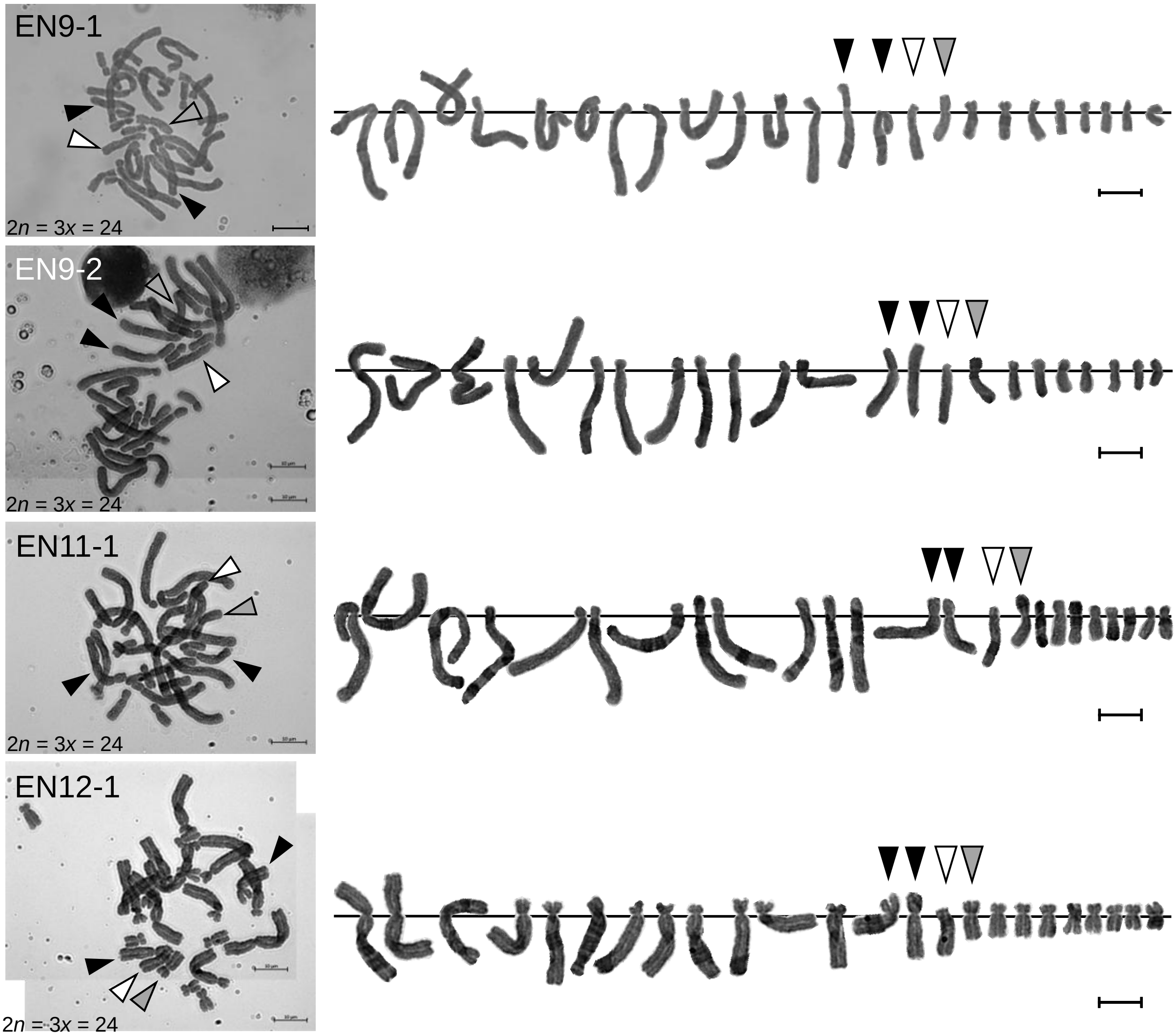


Fig. S3 Cont.
Representative chromosome images and karyograms of 16 endosperm-derived plantlets from each seed. Each arrowhead means the characteristic chromosome of *H*. *pauculifolius* (black) and *H*. *albiflos* (white and gray). Scale bars = 10 µm.
