# Supplementary material for: Endosperm culture-based allotriploid hybrid production from an interspecific cross of Haemanthus spp.: new insights into polyploidization and hybridization
Source: BMC Plant Biol. 2025 Feb 6;25:158. doi: 10.1186/s12870-025-06181-x (PMC11800442; doi:10.1186/s12870-025-06181-x)
Supplement: Supplementary file 8 — Additional file 8. Fig. S3. Representative chromosome images and karyograms of 16 endosperm-derived plantlets from each seed. Fig. S3 shows representative chromosome images and karyograms of 16 endosperm-derived plantlets from each seed. [file 12870_2025_6181_MOESM8_ESM.pdf]

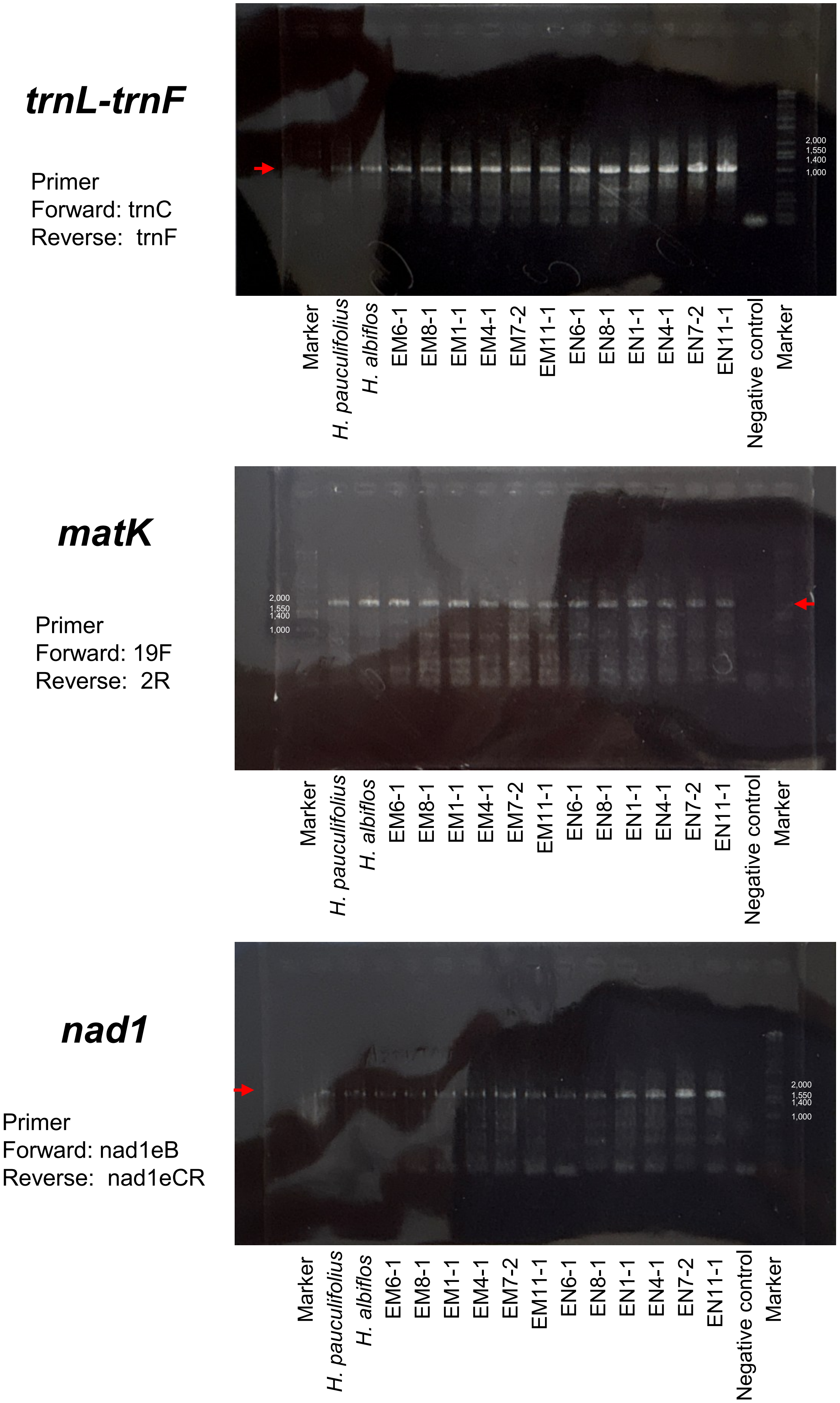

Fig. S4

Electrophoresis images showing the amplification of the *trnL-trnF* region, *matK* gene, and *nad1* gene in *Haemanthus pauculifolius*, *H. albiflos*, and embryo- and endosperm-derived plantlets.

The marker used was the Wide-Range DNA Ladder (50–10,000 bp). Arrows indicate the target DNA bands.
